# Pharmacologic inhibition of RBBP4/p300-mediated homologous recombination activity enhances glioblastoma sensitivity to temozolomide

**DOI:** 10.1093/noajnl/vdag141

**Published:** 2026-05-29

**Authors:** Josephine A Mapunda, Daniel J Laverty, Faisal Aziz, An Le, Junita Sangare, Lin Zhang, Zachary D Nagel, Jarrod B French, Jann N Sarkaria, Gaspar J Kitange

**Affiliations:** The Hormel Institute, University of Minnesota, Austin, Minnesota, USA; Department of Environmental Health, Harvard T.H. School of Public Health, Boston, Massachusetts, USA; Department of Chemistry, Lehigh University, Bethlehem, Pennsylvania, USA; The Hormel Institute, University of Minnesota, Austin, Minnesota, USA; The Hormel Institute, University of Minnesota, Austin, Minnesota, USA; The Hormel Institute, University of Minnesota, Austin, Minnesota, USA; Division of Biostatistics & Health Data Science, University of Minnesota, Minneapolis, Minnesota, USA; Department of Environmental Health, Harvard T.H. School of Public Health, Boston, Massachusetts, USA; The Hormel Institute, University of Minnesota, Austin, Minnesota, USA; Department of Radiation Oncology, Mayo Clinic, Rochester, Minnesota, USA; The Hormel Institute, University of Minnesota, Austin, Minnesota, USA

**Keywords:** DNA damage repair, glioblastoma, homologous recombination, p300, RBBP4, temozolomide

## Abstract

**Background:**

Upstream regulators of the homologous recombination (HR) repair pathway are promising targets for overcoming temozolomide (TMZ) resistance. We investigated whether pharmacologic inhibition of Retinoblastoma Binding Protein 4 (RBBP4)/p300-mediated HR activity by CCS1477 or NEO2734 sensitizes glioblastoma (GBM) to TMZ.

**Methods:**

Lentiviral-mediated shRNA was used to silence RBBP4 and EP300 (p300). A fluorescence-based multiplex flow-cytometric host cell reactivation assay was used to measure DNA repair activity, while TMZ-induced double-strand breaks (DSBs) were assessed using a comet assay and γ-H2AX foci formation. Cytotoxicity was monitored using an Incucyte device, and survival analysis was performed to evaluate efficacy in orthotopic tumor models. Drug distribution was assessed using liquid chromatography-mass spectrometry (LC-MS/MS) and acetylation of lysine 27 of histone H3 (H3K27Ac) immunofluorescence for target inhibition. Promoter occupancy was determined using chromatin immunoprecipitation.

**Results:**

GBM patient-derived xenograft tumors with high *RBBP4* expression showed elevated levels of 6 HR genes (*RAD51*, *RAD50*, *BRCA1*, *BARD1*, *BRIP1*, and *FIGNL1*), with *RAD51* and *BRIP1* correlating strongly with *RBBP4* and *p300*. Silencing *RBBP4* or *p300* decreased HR and microhomologous-mediated end joining (MMEJ) activity and delayed repair of TMZ-induced DSBs. CCS1477 and NEO2734 suppressed *RAD51* and *MYC* in a concentration-dependent manner, prolonged γ-H2AX foci, and enhanced TMZ sensitivity. Both compounds inhibited H3K27Ac and were detectable in orthotopic tumors by LC-MS/MS and significantly extended survival alone and in combination with TMZ.

**Conclusion:**

These findings suggest that the RBBP4/p300-axis is a key regulator of HR-mediated repair of TMZ-induced DSBs, and inhibition by either CCS1477 or NEO2734 may be beneficial as monotherapy, but further studies are needed to determine the benefit of combining these agents with TMZ.

Key Points

- RBBP4/p300 complex may modulate DNA repair activity by regulating the transcription of key HR genes.
- Disrupting RBBP4/p300 complex suppresses the resection-dependent DNA repair pathways, HR and MMEJ.
- CCS1477 or NEO2734 enhances TMZ sensitivity in vitro but modestly in orthotopic GBM PDXs.

Importance of the Study

Although the repair of TMZ-induced DNA double-strand breaks (DSBs) has been linked to homologous recombination (HR) activity in glioblastoma (GBM) cells, the upstream mechanisms regulating HR and the impact of pharmacologic inhibition of these mechanisms on TMZ sensitivity remain unclear. Here, we demonstrate that the RBBP4/p300 axis controls HR activity by regulating the expression of 6 key HR genes. We further show that targeting this HR-regulatory axis with the clinically relevant inhibitors CCS1477 and NEO2734 significantly sensitizes GBM cells to TMZ. Both compounds demonstrate the ability to penetrate the blood-brain barrier, distribute within orthotopic GBM tumors, and extend survival in tumor-bearing mice, with a modest but significant additional benefit when combined with TMZ. Collectively, these findings establish a foundation for future studies aimed at optimizing inhibition of the RBBP4/p300 complex and refining treatment schedules to enhance TMZ sensitization by CCS1477 and NEO2734.

Despite maximal surgical resection, radiation, and temozolomide (TMZ)-based chemotherapy, IDH1/2 wild-type glioblastoma (GBM) remains the most aggressive primary brain tumor, with a median survival of approximately 15 months for newly diagnosed patients.^1-3^ The high rate of GBM recurrence, which underlies this poor prognosis, is largely driven by the strong propensity of tumor cells to develop therapeutic resistance, including resistance to TMZ.^3-5^

One of the key mechanisms underlying resistance to TMZ is the ability of GBM cells to repair therapy-induced DNA damage.^6^^,^^7^ TMZ exerts its antitumor effects by inducing O6-methylguanine (O6-MG) DNA lesions, which, if unrepaired, cause replication stress-induced single-strand DNA breaks that eventually progress to DSBs.^8^^,^^9^ Intrinsic resistance to TMZ is largely controlled by the O6-methylguanine-DNA-methyltransferase (MGMT) repair protein, which removes O6-MG lesions in tumors with an unmethylated MGMT-promoter.^8-12^ In contrast, MGMT-promoter hypermethylated GBM cells can evade TMZ-induced cytotoxicity by activating DNA damage repair pathways, particularly the homologous recombination (HR) pathway, which helps cells recover from the stalled replication forks triggered by persistent O6-MG lesions.^13^ Accordingly, upregulation of HR repair genes is a poor prognostic factor in GBM patients.^14^ This highlights HR as an important contributor to acquired TMZ resistance and a potential therapeutic vulnerability.

Retinoblastoma Binding Protein 4 (RBBP4) is a chromatin remodeling protein that has recently emerged as an important link between epigenetic regulation and DNA repair genes in GBM and possibly in other human cancers.^15^^,^^16^ RBBP4 interacts with p300 to form a chromatin remodeling RBBP4/p300 complex, which is active in GBM cells. We recently reported that disruption of this complex by silencing RBBP4 or p300 downregulates the expression of 6 genes involved in the HR pathway, including *RAD51*, *BRCA1*, *and BRIP1*.^16^^,^^17^ However, whether the RBBP4/p300 complex directly modulates the levels of expression of these HR genes in GBM and the impact of this complex on HR activity in the context of the repair of TMZ-induced DSBs remain unelucidated.

Here, we demonstrate that the expression of *RBBP4* or *p300* strongly correlates with the expression of HR genes in GBM patient-derived xenograft (PDX) tumors. Moreover, perturbation of the RBBP4/p300 axis through shRNA-mediated silencing or pharmacologically by using the p300 inhibitor CCS1477 or dual p300/BRD4 inhibitor NEO2734 impairs this regulatory pathway, reduces HR activity, enhances TMZ sensitivity in GBM cells, and prolongs survival in orthotopic PDX models. These findings underscore the therapeutic potential of targeting the RBBP4/p300 complex to impair DNA repair proficiency, thereby overcoming GBM resistance to TMZ.

## Methods

### Cell Culture

Primary PDX cells from GBM43, GBM67, GBM8, and GBM22 were cultured as previously described.^18^

### Chromatin Immunoprecipitation and Chromatin Immunoprecipitation Sequencing

The standard chromatin immunoprecipitation (ChIP) and ChIP-sequencing (ChIP-seq) were done as previously reported.^16^ The additional information on the standard ChIP assay is available in online supplementary material, while the details on ChIP-seq analysis can be accessed in reference Kitange et al,^16^ and the data are available in GSE196383.

### Western Blot Analysis

Western blotting was performed according to the previously reported protocol.^15^ For detailed information regarding the primary and secondary antibodies used, please refer to the online supplementary material. Briefly, protein lysates were extracted using radioimmunoprecipitation assay buffer (Thermo Fisher Scientific, Waltham, MA). Proteins were separated by electrophoresis on 4%-12% polyacrylamide gels and transferred to a nitrocellulose (or polyvinylidene fluoride (PVDF)) membrane for at least 1 h. The membranes were incubated with primary antibodies (1:1000 dilution) with shaking overnight at 4 °C. Following primary incubation, membranes were washed 3 times for 5 min each in Tris-buffered saline with Tween 20 (T-TBS), followed by a 1-h incubation with secondary antibodies at room temperature. After 3 additional 5-min washes in T-TBS, protein bands were detected using chemiluminescence reagents (Thermo Fisher Scientific) and imaged with an Azure 600 imager (Azure Biosystems, Inc., Dublin, CA). Where applicable, band intensity was quantified using ImageJ software. All Western blots were performed in at least 3 independent biological replicates.

### In Vitro Cytotoxicity Assay

Primary cultured PDX cells were plated in 96-well plates (1000-2000/well) and allowed 24-48 h attachment at standard humidified culture conditions (37 °C and 5% CO_2_). Then, cells were exposed to graded concentrations of CCS1477 (TargetMol Chemicals Inc., Wellesley Hills, MA), NEO2734 (TargetMol), TMZ (MilliporeSigma, Burlington, MA), and combined CCS1477/TMZ or NEO2734/TMZ. The control cells were treated with DMSO. The growth of the control and treated cells was continuously monitored using an Incucyte SX5 Device (Sartorius Inc., Ann Arbor, MI). To complement the Incucyte data, experiments were repeated using the CyQuant Cell Proliferation Assay (Thermo Fisher Scientific). Briefly, cells were plated in 96-well plates (2000-3000 cells/well) and maintained for at least 24 h before treatment with CCS1477, NEO2734, TMZ, or the indicated combinations. Cytotoxicity was evaluated according to the manufacturer’s protocol, and fluorescence was measured using a Tecan Infinite 200 Pro multiplate reader (Tecan Group Ltd., Männedorf, Switzerland). All experiments were conducted in triplicate and independently repeated 3 times.

### Gene Knockdown

Lentiviral-mediated shRNA gene knockdown was performed as we reported previously.^16^ Briefly, monolayer cells were transduced with lentiviral particles in the presence of polybrene (10 µg/mL), and antibiotic selection was initiated 48 h after transduction. Effective knockdown was assessed by Western blot analysis, and shRNA constructs achieving ≥90% inhibition were selected for subsequent experiments.

### DNA Repair Activity Reporter Assay

The DNA repair activity was measured using fluorescence-based multiplex flow-cytometric host cell reactivation assays (FM-HCR) for repair of DSBs by HR and MMEJ as described previously.^19-21^

### Comet Assay

GBM22 and GBM43 cells expressing control non-targeting shRNA (shNT), shRBBP4, or shp300 were grown for 48 h under standard cell culture conditions. Cells were then treated with either vehicle (DMSO) or TMZ. After 72 h, cells were harvested by gentle dissociation, washed with ice-cold PBS, and resuspended at a concentration of 1 × 10^5^ cells/mL. The neutral comet assay was performed using the Enzo Comet SCGE assay kit (Cat. ADI-900-166) according to the manufacturer’s instructions (Enzo Biosciences Inc., Farmingdale, NY). Slides were imaged using an LSM900 confocal microscope equipped with a 20× objective (Zeiss LSM 900 with Airyscan detector, Zeiss, Germany). Comets were visualized under green fluorescence (FITC filter), and images were captured using identical exposure settings. A minimum of 100 comets per sample was analyzed using the OpenComet plugin in ImageJ software. The tail moment (product of tail length and % DNA in the tail) was calculated and used as a quantitative measure of DNA damage.

### γ-H2AX DNA Damage Foci

GBM22 and GBM43 cells were grown for 48 h on coverslips. Cells were treated with either vehicle (DMSO), 800 nM CCS1477, 800 nM NEO2734, 20µM TMZ with and without CCS1477, or NEO2734. After 72 h incubation, cells were fixed with 4% paraformaldehyde. Irradiated cells (2 Gy) were used as a positive control for γ-H2AX. The immunofluorescence (IF) staining was conducted as previously described.^16^ The antibodies used are shown in online supplementary ­material. The staining was analyzed with a confocal microscope (Zeiss LSM 900 with Airyscan detector, Zeiss, Germany). For γ-H2AX foci quantification, at least 200 cells with ≥25 foci/nuclei were analyzed for each condition.

### In Vivo Distribution and Target Inhibition by CCS1477 and NEO2734 Compounds

To evaluate the brain penetration potential of CCS1477 and NEO2734, the plasma, brain, and tumor distribution studies were performed in female FOXN1 nude mice bearing GBM43 orthotopic tumors. Animals were randomized into 3 treatment groups (*n* = 9/group) and orally administered either a placebo, CCS1477 (20 mg/kg), or NEO2734 (10 mg/kg) once daily for 5 consecutive days. Mice were sacrificed at 3 different time points (3 mice/time point) following the last dose: 30 min, 2 h, and 4 h. Blood was collected by cardiac puncture for serum separation, while whole brains were rapidly dissected to isolate both tumor tissue and contralateral non-tumor-bearing brain. All tissues were snap-frozen in liquid nitrogen and stored at −80 °C until further processing. Drug concentrations in serum, tumor, and non-tumor brain tissue were quantified using liquid chromatography-mass spectrometry (LC-MS/MS) as detailed in online ­supplementary material.

To complement LC-MS/MS data, the effects of CCS1477 and NEO2734 on acetylation of lysine 27 of histone H3 (H3K27Ac) were assessed 2 h after the last dose as described in online supplementary material.

### Evaluation of Drug Efficacy in Orthotopic GBM Xenografts

All animal studies were approved by the University of Minnesota Institutional Animal Care and Use Committee (IACUC). To evaluate the efficacy of CCS1477 and NEO2734 on TMZ sensitivity, mice bearing GBM43 orthotopic tumors were randomized in 6 treatment arms (10 mice/arm). Tumor-bearing mice were orally treated with either placebo (Ora-Plus), 20 mg/kg CCS1477, 10 mg/kg NEO2734, 50 mg/kg TMZ, or TMZ in combination with either CCS1477 or NEO2734 daily for 5 days and then monitored until moribund. Mice body weights were recorded daily, and a loss of ≥25% was considered indicative of end-stage tumor burden or toxicity, warranting euthanasia.

### Statistical Analysis

Pearson correlation coefficient was computed to assess the association of the expression levels of *RBBP4* or *p300* with those of the HR genes. In vitro drug sensitivity studies were conducted in triplicate for 3 independent experiments. The Student *t*-test was used to evaluate differences across treatment groups, and *P* value < .05 were considered statistically significant. The drug synergy was evaluated using McSynergy II software (University of Michigan, MI), and a synergy score ≥100 was considered significant. Animal survival probabilities were estimated using the Kaplan-Meier method, and the log-rank test was used to compare the survival curves between groups, with *P* < .05 considered statistically significant.

## Results

### RBBP4 Transcripts Level Correlates With HR Repair Gene Expression in GBM PDXs

**Figure 1.**
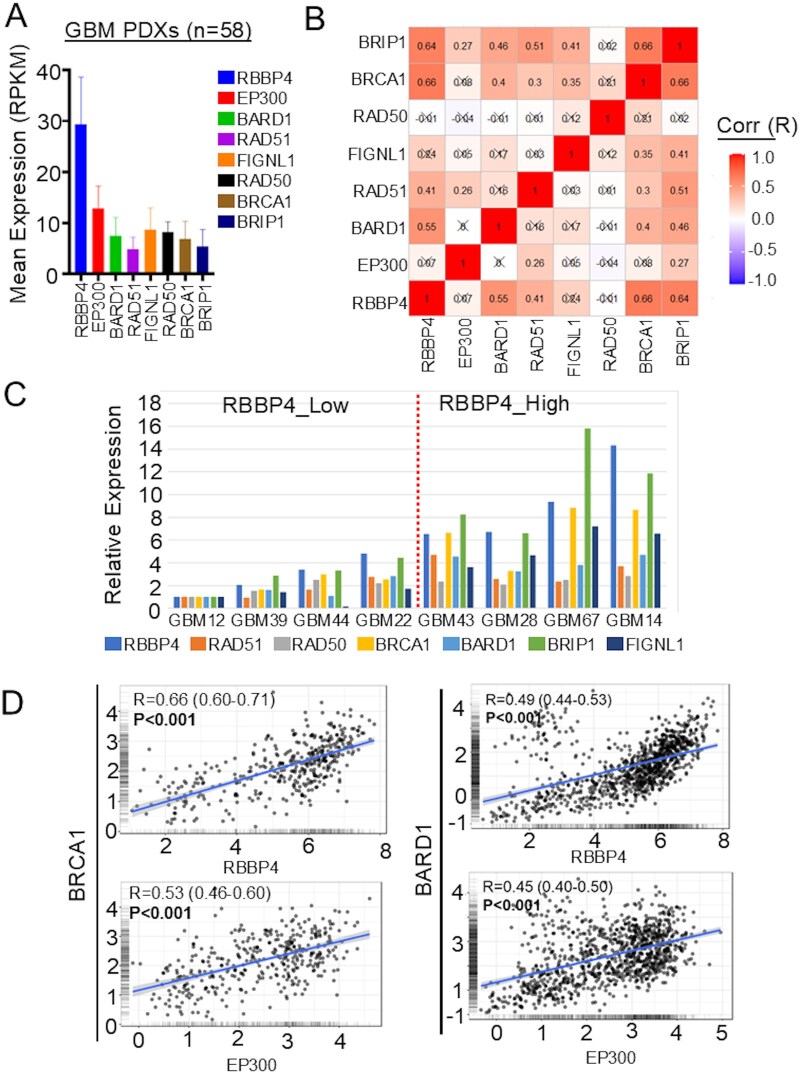
Expression and correlation of *RBBP4* and *p300* with 6 HR genes in GBM PDX models. Mayo Clinic GBM PDX RNA sequencing data were obtained from cBioPortal for Cancer Genomics. (A) Bar graphs display the mean expression levels of DNA repair genes in 58 GBM PDXs. Error bars represent the SD. (B) Pearson correlation coefficients (R) across GBM PDX samples. (C) GBM PDX cells stratified by their *RBBP4* expression levels and compared to the relative expression of 6 DNA repair genes. (D) Scatter plots showing the correlation between *RBBP4* and *p300* with HR gene *RAD51* and *BARD1*. Pearson correlation coefficients (R) and *P* value are displayed in each panel. GBM, glioblastoma; HR, homologous recombination; PDX, patient-derived xenograft; RBBP4, Retinoblastoma Binding Protein 4.

To investigate the expression profile of *RBBP4* and HR repair genes across GBM patients, we acquired and analyzed RNA sequencing data of the Mayo Clinic GBM PDX cohort available in the cBioPortal for Cancer Genomics (https://www.cbioportal.org/). Among the 58 PDX analyzed, we observed a robust expression of *RBBP4* and *EP300 (p300)* alongside key HR repair genes, including *BRCA1*, *BRIP1*, *BARD1*, *RAD50*, *RAD51*, and *FIGNL1* (Figure 1A). The expression of RBBP4 and the 6 HR gene transcripts for all PDX models is displayed in Figure S1. The Pearson correlation analysis revealed the strongest positive correlation between *RBBP4* and *BRCA1* (*P* < .0001), followed by *BRIP1* (*P* < .0001), *BARD1* (*P* < .0001), and lastly *RAD51* (*P* = .0012) (Figure 1B). In contrast, no significant correlations were observed between *RBBP4* and *RAD50* (*P* = .97) or *FIGNL1* (*P* = .067). Notably, *EP300* expression displayed a significant positive correlation only with *RAD51* (*P* = .0320) and *BRIP1* (*P* = .0098) (Figure 1B). To determine whether *RBBP4* expression may directly modulate HR gene expression, we stratified 8 GBM PDXs into *RBBP4*^Low^ (*n* = 4) and *RBBP4*^High^ (*n* = 4) groups and performed qRT-PCR analysis. As shown in Figure 1C, the *RBBP4*^High^ PDXs exhibited substantially higher transcripts of RBBP4/p300-regulated HR genes compared with the *RBBP4*^Low^ PDXs. These observations were further supported by the correlation analyses using GBM patient datasets from GlioVis (https://gliovis.bioinfo.cnio.es/), which confirmed a direct correlation between *RBBP4* and *p300* with the 6 HR genes (Figure 1D;  Figure S2). Moreover, high *RBBP4* expression correlated with poor GBM patient outcome (Figure S3). Collectively, these findings underscore the role of the RBBP4/p300 complex in regulating the expression of key 6 HR repair genes in GBM and may influence patient response to therapy.

### High RBBP4 and H3K27Ac Tag Density Within Promoters of HR Genes in High RBBP4-Expressing GBM PDXs

**Figure 2.**
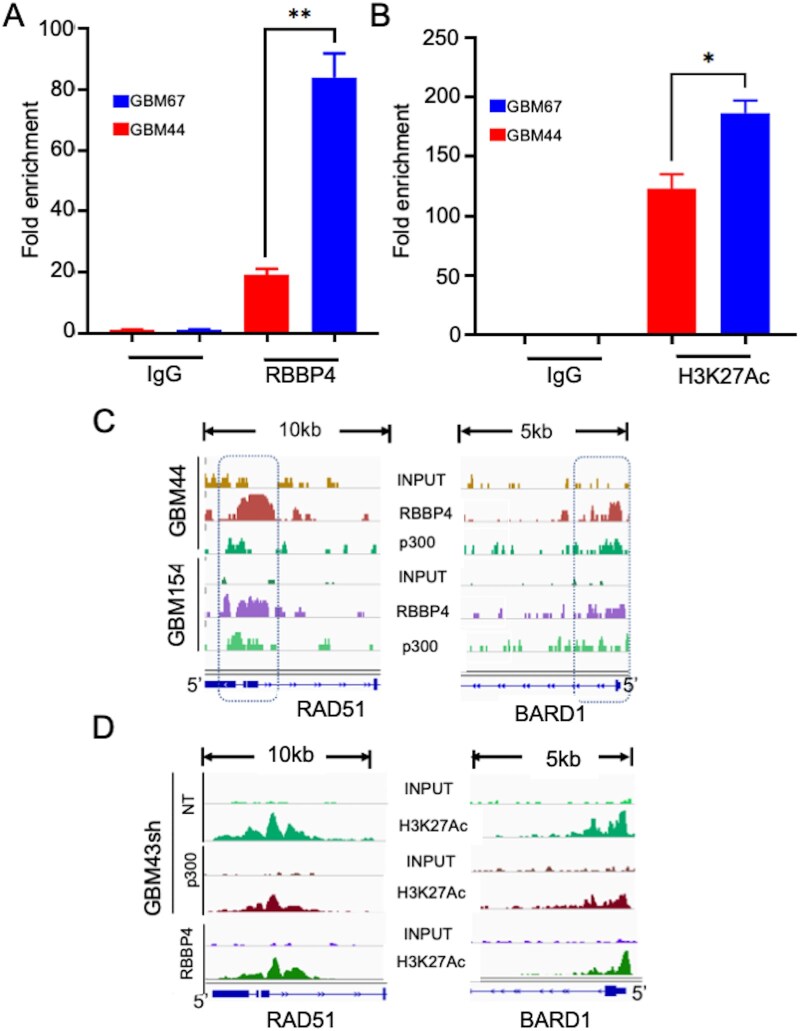
Promoter recruitment of RBBP4 and H3K27Ac in association with RBBP4 expression status. Tissue ChIP analysis in GBM44 (RBBP4^Low^-expressing cells) and GBM67 (RBBP4^High^-expressing cells) to assess the fold enrichment levels of (A) RBBP4 and (B) H3K27Ac relative to IgG control at the *RAD51* locus. (*P < .01 and **P < .05). Data represents the mean ± SEM of 3 independent experiments. (C) ChIP-seq analysis of GBM44 and GBM154 PDX cells showing RBBP4 and p300 binds at the promoter regions of *RAD51* (left) and *BARD1* (right). Genomic regions of interest are highlighted with dashed boxes. (D) Representative ChIP-seq analysis showing knockdown of *RBBP4* and *p300* reduces the enrichment of H3K27Ac within the promoter region of *RAD51* (left) and *BARD1* (right) loci in GBM43 cells. ChIP-seq, chromatin immunoprecipitation sequencing; GBM, glioblastoma; H3K27Ac, acetylation of lysine 27 of histone H3; PDX, patient-derived xenograft; RBBP4, Retinoblastoma Binding Protein 4.

To evaluate whether *RBBP4* transcript levels influence RBBP4 protein recruitment and histone acetylation status at HR gene promoters, we performed tissue ChIP-qPCR analysis on the RAD51 promoter region. Two GBM PDX models were examined: GBM44 (*RBBP4*^Low^) and GBM67 (RBBP4^High^) (see Figure 1C). Compared with their respective IgG controls, *RBBP4*^High^ GBM67 exhibited significantly higher RBBP4 enrichment at the *RAD51* gene promoter than *RBBP4*^Low^ GBM44 (Figure 2A). Similarly, H3K27Ac enrichment at the *RAD51* promoter was markedly higher in *RBBP4*^High^ GBM67 than in *RBBP4*^Low^ GBM44 (Figure 2B), suggesting that elevated RBBP4 expression correlates with increased histone acetylation at the *RAD51* promoter and at other HR gene promoters regulated by the RBBP4/p300 complex.

To corroborate the ChIP-qPCR data, we revisited our previous independently generated ChIP-seq datasets from GBM44 and GBM154 (GSE196383). We assessed whether RBBP4 and p300 co-occupy the promoter regions of these RBBP4/p300-regulated HR genes. As shown in Figure 2C, both GBM44 and GBM154 exhibited strong RBBP4 and p300 tag enrichment at the *RAD51* (left panel) and *BARD1* (right panel) promoter regions. This co-binding pattern supports the involvement of the RBBP4/p300 complex in the transcriptional activation of HR repair genes. To validate these observations, we queried the same ChIP-seq dataset (GSE196383) to determine whether shRNA-mediated silencing of *RBBP4* or *p300* suppresses H3K27Ac, a histone mark associated with transcriptionally active chromatin, at the promoter/enhancer regions of *RAD51* and *BARD1*. In cells expressing shNT, H3K27Ac was highly enriched at both *RAD51* (Figure 2D, left panel) and *BARD1* (Figure 2D, right panel) promoter loci. In contrast, knockdown of either *RBBP4* or *p300* significantly reduced H3K27Ac levels, confirming that RBBP4 and p300 mediate the histone acetylation mark at the promoters/enhancers of *RAD51*, *BARD1*, and other RBBP4/p300-regulated HR genes. Collectively, these findings establish that the RBBP4/p300 complex regulates histone acetylation at HR gene promoters, including *RAD51* and *BARD1*, thereby promoting their expression.

### Disrupting RBBP4/p300 Complex Suppresses HR and MMEJ Proficiency in GBM

**Figure 3.**
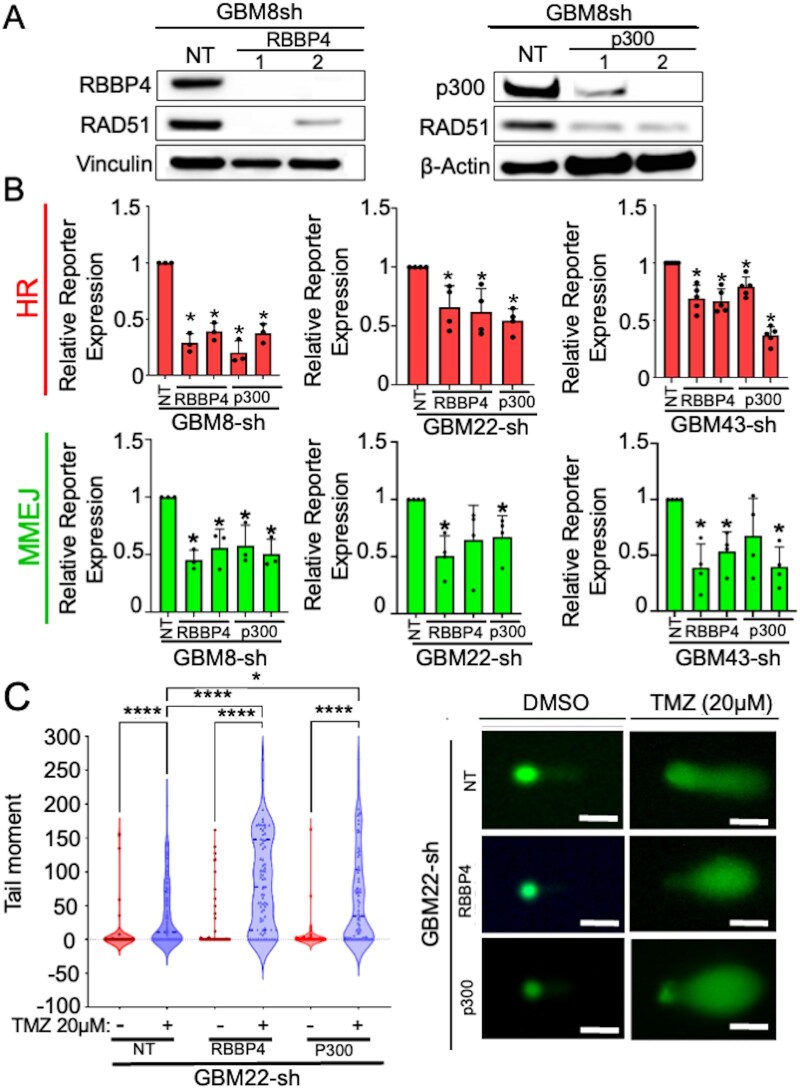
Impact of silencing *RBBP4* or *p300* on DNA repair pathway choice. (A) Western blot showing reduced levels of RAD51 protein following the knockdown of RBBP4 (left) or p300 (right). (B) DNA repair reporter assays in GBM8, GBM22, and GBM43 cells showing reduction in both HR repair activity (upper panel) and MMEJ repair activity (lower panel) upon knocking down of either RBBP4 or p300. In each panel, reporter expression in the lines depleted for RBBP4 or p300 is normalized to reporter expression in the non-targeting control. Data represents the mean ± SEM of 3 independent experiments. (C) Violin plots (left panel) indicating increased DNA damage after treatment of GBM22 (shNT, sh*RBBP4*, and sh*p300*) either DMSO or 20 µM of TMZ. Representative comet assay images showing increased DNA fragmentation in sh*RBBP4* and sh*p300* GBM22 cells treated with TMZ (right panel). Scale bar = 10 µm. Statistical significance is indicated as *P < .05, ****P < .0001. GBM, glioblastoma; RBBP4, Retinoblastoma Binding Protein 4; shNT, non-targeting shRNA; TMZ, temozolomide.

We next sought to determine whether depletion of *RBBP4* or *p300* impairs DNA repair activity in GBM cells. To this end, we silenced either *RBBP4* or *p300* in GBM8, GBM22, and GBM43 PDX cells and evaluated the impact on HR, non-homologous end joining (NHEJ), and MMEJ repair activities. Consistent with our previous report,^16^ depletion of either *RBBP4* or *p300* significantly reduced RAD51 protein levels in GBM8 cells (Figure 3A). Similar results were observed in GBM22 and GBM43 cells (Figure S4). We then assessed the effect of *RBBP4* and *p300* depletion on DNA repair efficiency in GBM8, GBM22, and GBM43 cells using an FM-HCR assay focusing on HR, NHEJ, and MMEJ pathways.^20^ Silencing *RBBP4* or *p300* significantly suppressed HR activity in all 3 PDX models (Figure 3B, upper panels), as well as MMEJ activity (Figure 3B, lower panels). In contrast, no significant changes were observed in classical NHEJ activity upon *RBBP4* or *p300* knockdown (Figure S5), indicating that the RBBP4/p300 complex may selectively regulate DNA resection-dependent DSB repair pathways, HR, and MMEJ.

To demonstrate that reduced repair proficiency leads to accumulation of DSBs, we performed a neutral comet assay to evaluate the effect of *RBBP4* or *p300* silencing on TMZ-induced DSBs. We observed a significant increase in tail moments in *RBBP4*- and *p300*-deficient cells compared with controls (GBM22 shNT-TMZ vs GBM22 shRBBP4, *P* < .01; GBM22 shNT-TMZ vs GBM22 shp300, *P* = .01) (Figure 3C, left panel; Figure S6), indicating an accumulation of TMZ-induced DSBs.^22^ Together, these findings demonstrate that perturbation of the RBBP4/p300 complex impairs DNA repair efficiency, resulting in the accumulation of TMZ-induced DSBs.

### Inhibiting RBBP4/p300-HAT Activity by CCS1477 and NEO2427 Sensitizes GBM Cells to TMZ In Vitro

**Figure 4.**
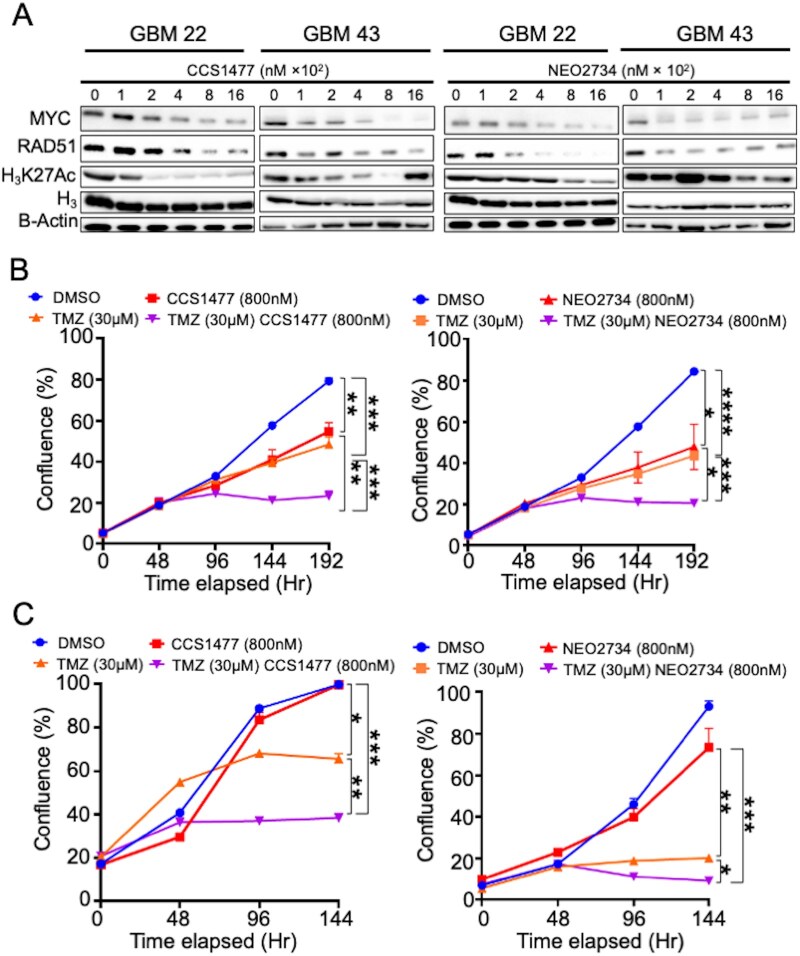
Inhibition of p300 sensitizes GBM cells to TMZ. (A) Western blot showing a dose-dependent reduction in RAD51 protein expression, C-MYC, and global H3K27Ac in both GBM22 (left panel) and GBM43 (right panel) cells. (B and C) Primary (C) GBM22 and (D) GBM43 were plated in 96-well plates and treated with the indicated concentrations of CCS1477, NEO2734, TMZ, and CCS1477 + TMZ or NEO2734 + TMZ, and growth was monitored using an Incucyte Live Cell monitoring device and reported in percent confluence. Statistical significance is indicated as *P < .05, **P value < .01, *** *P* < .001, ****P value < .0001. GBM, glioblastoma; TMZ, temozolomide.

After providing evidence that the RBBP4/p300 complex regulates the 6 HR repair activity in GBM cells, we next wanted to determine whether pharmacological inhibition of RBBP4/p300-HAT activity will enhance TMZ sensitivity in GBM cells. To that end, we first performed target inhibition in vitro studies by treating both parental GBM22 and GBM43 cells with increasing concentrations of either CCS1477 (p300/CBP inhibitor) or NEO2734 (dual BRD4/p300 inhibitor). Western blot analysis revealed that, like our sh*RBBP4* and sh*p300* findings shown above in Figure 3A, Kitange et al,^16^ and Mladek et al,^17^ both inhibitors led to a dose-dependent reduction in RAD51 expression, with the strongest effect observed at higher concentrations in both GBM22 and GBM43 cells (Figure 4A). Furthermore, both C-MYC and global H3K27Ac levels were significantly reduced in both cell lines, confirming effective inhibition of the RBBP4/p300-HAT activity by both inhibitors (Figure 4A). To evaluate whether the pharmacological inhibition of RBBP4/p300-HAT activity by CCS1477 or NEO2734 influences the sensitivity of GBM cells to TMZ, we treated 2 GBM PDX cells (MGMT-unmethylated GBM43 and MGMT-methylated GBM22) with an increasing dose of either drug alone or in combination with TMZ. Interestingly, the MGMT promoter methylated GBM22 cells were sensitive to both CCS1477 and NEO2734 alone (DMSO vs CCS1477, *P* = .01; DMSO vs NEO2734, *P* = .03), and there was a significant additional growth suppression by combining TMZ with either CCS1477 or NEO2734 (TMZ vs CCS1477 + TMZ, *P* < .01; TMZ vs NEO2734 + TMZ, *P* < .01; Figure 4B). Similar results were observed in the MGMT promoter unmethylated GBM43 (DMSO vs CCS1477, *P* < .01; DMSO vs NEO2734, *P* < .01; TMZ vs CCS1477 + TMZ, *P* < .01; TMZ vs NEO2734 + TMZ, *P* < .01) (Figure 4C). To complement our Incucyte-based cell confluence data, we performed similar experiments using the CyQuant Cell Proliferation Assay (Thermo Fisher Scientific, Waltham, MA) and used the resulting data to analyze drug combination synergy. Consistent with the Incucyte findings, the CyQuant data demonstrated a significant sensitization of GBM22 and GBM43 cells to TMZ, and this effect was synergistically significant (Figure S7). These findings demonstrated a significant sensitization of TMZ by the clinically relevant CCS1477 and NEO2477, further supporting RBBP4/p300-HAT inhibition as a strategy to sensitize GBM to TMZ.

### CCS1477 and NEO2734 Enhance TMZ-Induced DNA Damage and Apoptosis

**Figure 5.**
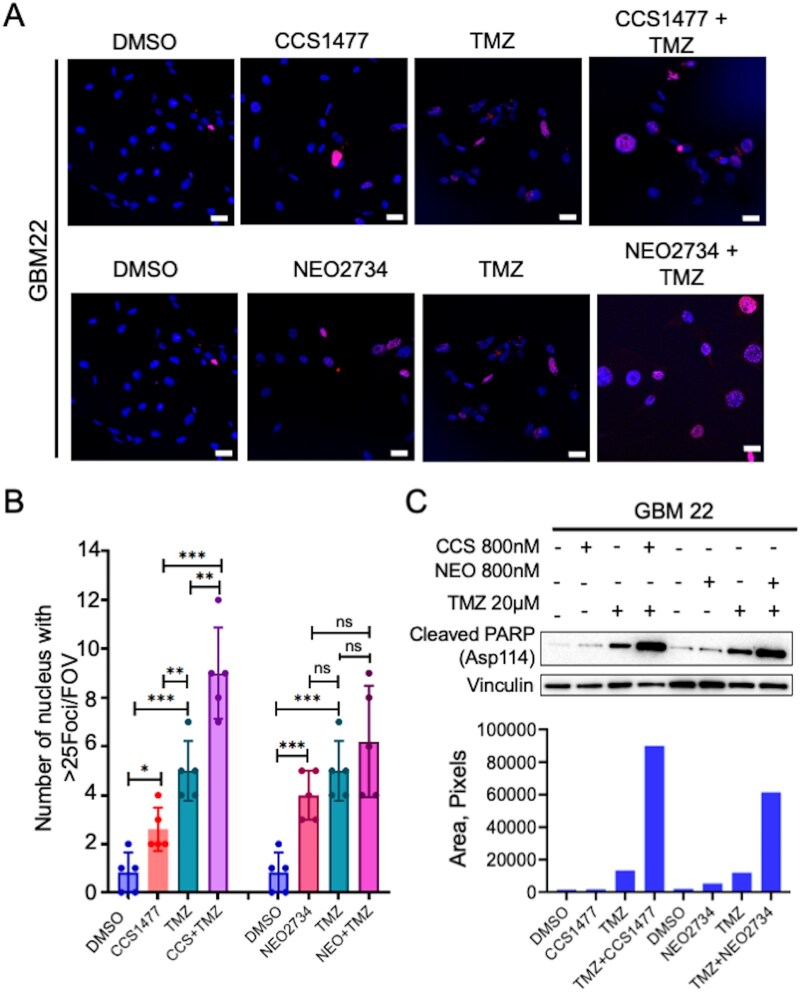
Pharmacological inhibition of p300 by CCS1477 or p300/BRD4 by NEO2734 enhances TMZ-induced DSBs and apoptosis in GBM cells. (A) Evaluation of γ-H2AX foci in GBM22 cells treated with CCS1477, NEO2734, TMZ, and CCS1477 + TMZ or NEO2734 + TMZ. γ-H2A foci were detected using immunofluorescence staining performed 72 h later. (B) The number of nuclei with ≥25 γ-H2AX foci was counted and graphed. (C) Western blot and the corresponding band quantification showing an increase in cleaved PARP (Asp114) levels in both GBM22 cells upon 72 h treatment with the indicated concentrations of CCS1477, NEO2734, TMZ, and CCS1477 + TMZ or NEO2734 + TMZ. Magnification bar = 20 µm. Statistical significance is indicated as *P < .05, **P < .01, ***P < .001. DSBs, double-strand breaks; GBM, glioblastoma; TMZ, temozolomide.

As an initial step toward understanding the mechanism through which CCS1477 and NEO2734 inhibitors sensitize GBM cells to TMZ, we directly assessed the repair kinetics of TMZ-induced DSBs using γ-H2AX foci resolution. Treatment with CCS1477 or NEO2734 alone induced a modest but significant increase in γ-H2AX signal in GBM22 cells (Figure 5A). Notably, as shown in Figure 5B, the combination of TMZ with CCS1477 led to a strong accumulation of γ-H2AX foci, significantly exceeding the levels observed with CCS1477 or TMZ alone (*P < .05 to ***P < .001). In contrast, co-treatment with TMZ and NEO2734 did not further elevate γ-H2AX foci beyond those seen with TMZ alone, which could be explained by the wide SD observed in the combined treatment. Similar results were confirmed in GBM43 cells (Figure S8A and B). Consistent with increased cytotoxicity, western blot analysis revealed increased levels of cleaved PARP (Asp114) in GBM22 (Figure 5C, left panel) and GBM43 cells (Figure S8C) following combined treatment with CCS1477 or NEO2734 and TMZ, indicating enhanced apoptotic cell death.

### CCS1477 and NEO2734 Increase the Survival and Enhances TMZ Sensitivity In Vivo

**Figure 6.**
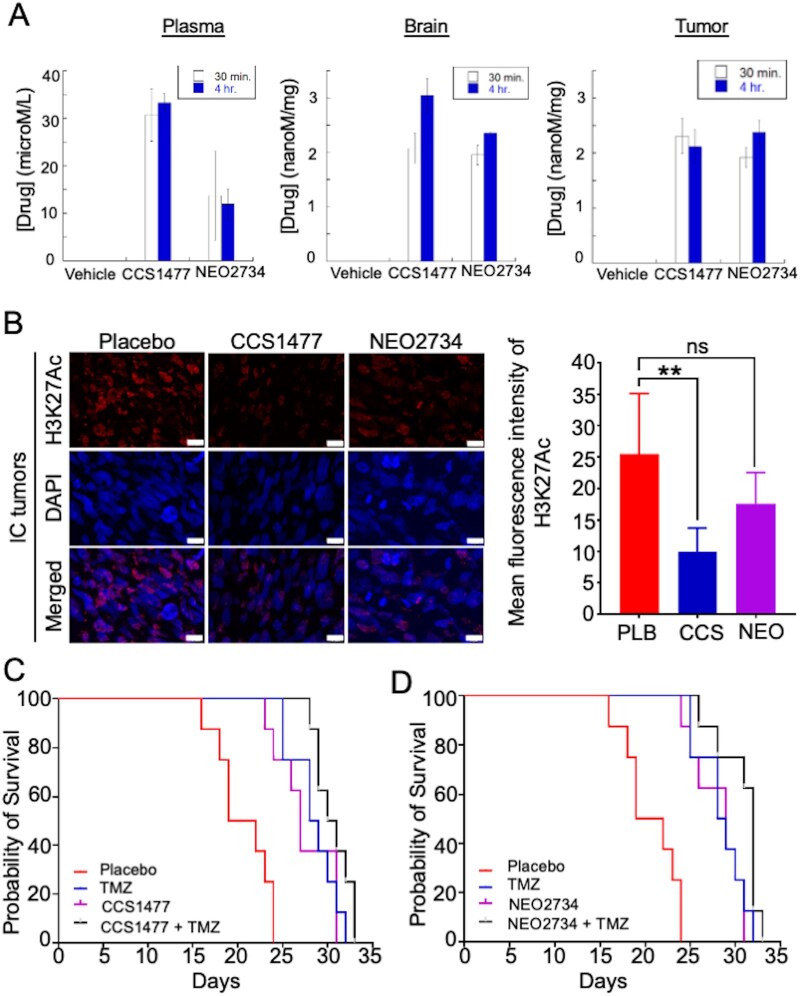
Effect of p300 inhibition on TMZ sensitivity in vivo. (A) Bar graphs displaying the distribution of CCS1477 and NEO2734 in the plasma, non-tumor brain, and tumor was detected by LC-MS/MS. (B) Representative images showing H3K27ac in mice bearing GBM43 IC tumors after treatment with placebo, 20 mg/kg CCS1477, or 10 mg/kg NEO2734 once daily for 5 days, with brains harvested 2 h after the last dose (scale bars = 20 µm). Bar graphs indicate the mean fluorescence intensity between the different treatment groups. (C and D) Kaplan-Meier plots showing survival of mice carrying GBM43 orthotopic tumors following completion of a 5-day treatment with (C) placebo, 50 mg/kg TMZ, 20 mg/kg CCS1477, and in combination with TMZ or (D) placebo, 10 mg/kg NEO2734, and in combination with TMZ. The placebo- and TMZ-treated arms are common for panels C and D. Survival was recorded based on the time to reach moribund state. The median survivals were as follows: placebo = 20.5 days vs TMZ = 28.50 days (*P* < .001), vs CCS1477 = 27 days (*P* < .001), and vs NEO2734 = 29 days (*P* < .001). Co-treatment with TMZ further improved survival, with a modest but significant benefit (median survival: CCS1477 + TMZ =27 days vs 30.5 days, *P* = .045) and in NEO2734 + TMZ-treated mice (median survival: 29 days vs 32 days, *P* = .027) (C and D). LC-MS/MS, liquid chromatography-mass spectrometry; TMZ, temozolomide.

Considering these promising in vitro results, we next sought to determine whether CCS1477 and NEO2734 can cross the blood-brain barrier (BBB) and achieve measurable distribution within GBM orthotopic tumors. To assess this, animals bearing orthotopic GBM43 tumors were orally treated once daily for 5 days with either 20 mg/kg CCS1477 or 10 mg/kg NEO2734. Serum, non-tumor brain, and tumor tissues collected at 30 min and 4 h after the final dose were further studied. LC-MS/MS analysis demonstrated robust systemic exposure and detectable concentrations of both CCS1477 and NEO2734 in plasma, non-tumor brain, and within tumor tissue (Figure 6A; Figure S9). Drug levels in non-tumor brain and tumor-bearing brain were normalized to tissue weight, and the results are shown in Figure 6A (middle and right panels). These findings confirm that CCS1477 and NEO2734 can penetrate intracranial tumors, likely reaching pharmacologically relevant concentrations.

Having established tumor drug exposure, we next evaluated target inhibition effects in both orthotopic and flank tumors. For this, we analyzed intracranial tumor samples from 2 mice per treatment group. For each mouse, 5 tissue sections were examined, resulting in a total of 10 sections per group included in the analysis. IF staining of intracranial GBM43 tumors revealed a significant reduction in H3K27Ac levels following CCS1477 treatment (*P* < .05), whereas NEO2734-treated tumors showed a less pronounced but still measurable reduction (Figure 6B). Importantly, flank tumors treated under the same dosing regimen also displayed a comparable decrease in H3K27Ac (Figure S10; *P* < .01), serving as a control for systemic target engagement outside the brain. The observed differences in H3K27Ac levels between mice bearing flank vs intracranial tumors after treatment with CCS1477 or NEO2734 may reflect the influence of the BBB and further confirm on-target inhibition of RBBP4/p300-HAT activity in vivo, which is consistent with our LC-MS/MS data.

Lastly, to evaluate whether CCS1477 or NEO2734 could potentiate the therapeutic effects of TMZ in vivo, GBM43 cells were stereotaxically implanted into the brains of mice. Ten days after tumor induction, mice were randomized into 6 treatment arms (10 mice/arm) and treated orally for 5 consecutive days with either placebo, 20 mg/kg CCS1477, 10 mg/kg NEO2734, 50 mg/kg TMZ, or a combination of 50 mg/kg TMZ with 20 mg/kg CCS1477 or 10 mg/kg NEO2734. Mice were monitored until they reached a moribund state, at which point they were sacrificed and survival data recorded. The initial follow-up identified no significant treatment-related weight loss (Figure S11). Treatment with TMZ with and without either CCS1477 (Figure 6C) or NEO2734 (Figure 6D) significantly prolonged median survival compared with placebo (TMZ vs placebo: *P* < .001; CC1477 vs placebo: *P* < .001; and NEO2734 vs placebo: *P* < .001 by log-rank test). The median survival was 20.5 days for placebo vs 28.5 days for TMZ, 27 days for CCS1477, and 29 days for NEO2734. Co-treatment with TMZ further improved survival, with a modest but significant benefit observed in CCS1477 + TMZ-treated mice (*P* = .045) with median survival to be 27 days vs 30.5 days, respectively, and in NEO2734 + TMZ-treated mice (*P* = .027) with median survival to be 29 days vs 32 days, respectively (Figure 6C and D). Collectively, these results demonstrate that both CCS1477 and NEO2734 penetrate the brain and provide preliminary support for further preclinical evaluation of these compounds in a larger cohort of GBM models.

## Discussion

Although the repair of DSBs by the HR machinery plays a critical role in TMZ resistance in GBM,^19^^,^^23^ the upstream epigenetic mechanisms regulating the transcription of HR genes has remained poorly understood. Here, we provide evidence that the RBBP4/p300 regulatory axis coordinately controls the transcription of 6 key HR genes, including RAD51 and BARD1, and that perturbation of this complex suppresses HR proficiency, delays DSB repair, and sensitizes GBM cells to TMZ. Moreover, we demonstrate for the first time the therapeutic potential of CCS1477 and NEO2734 in GBM through inhibition of RBBP4/p300-mediated HAT activity.

Understanding the upstream mechanisms that promote DSB repair by enhancing HR activity is essential for overcoming TMZ resistance. This is particularly important for patients with MGMT-promoter hypermethylated GBM tumors, where the absence of MGMT expression makes HR the primary pathway for repairing TMZ-induced DSBs.^8^ Our previous work demonstrated that RBBP4 is critical for maintaining the expression of DNA repair genes in GBM.^2^ We subsequently showed that RBBP4 interacts with p300 to form a regulatory epigenetic axis that sustains transcription of DNA repair genes through promoter and enhancer histone acetylation.^17^ Here, we extended these findings by showing that GBM PDX tumors with high *RBBP4* expression also express high levels of the 6 key HR gene transcripts. Specifically, *RBBP4* expression significantly correlated with 4 (*BARD1*, *RAD51*, *BRCA1*, and *BRIP1*) out of the 6 RBBP4/p300-regulated HR genes, whereas *p300* significantly correlated with only 2 genes (*RAD51* and *BRIP1*). Although these findings suggest the possibility that p300 may regulate a subset of HR genes independent of its interaction with RBBP4, this scenario is less likely. We previously demonstrated that silencing either *RBBP4* or *p300* suppressed all 6 HR genes, including *RAD51*, and produced a similarly global reduction of H3K27Ac across the promoters and enhancers of these genes.^16^^,^^17^ A study by Li et al^24^ reported that RBBP4 regulates the expression of the MRN complex to promote the repair of DSBs induced by chemotherapy in GBM. However, while we also identified RAD50 (a member of the MRN complex) as a target gene regulated by the RBBP4/p300 complex, our current study identified 5 additional HR genes regulated by this complex. These differences may be attributed not only to their specific focus on RBBP4 alone, but also to the distinct genetic backgrounds of the cells; Li et al utilized established cell lines, whereas we employed primary PDX cells. By investigating both RBBP4 and p300, the current study establishes the RBBP4/p300 complex as a potential master regulator of HR genes in GBM, and potentially in other human cancers.

Interestingly, disruption of the RBBP4/p300 complex also suppressed the activity of the resection-dependent MMEJ repair pathway, which shares the initial end resection step with HR.^25-27^ In contrast, no impact was observed on the proficiency of NHEJ, a resection-independent repair pathway.^25^ Both the HR and MMEJ pathways have been implicated in acquired TMZ resistance.^19^^,^^20^^,^^23^ Thus, these findings underscore the relevance of the RBBP4/p300 complex as a specific target for sensitizing GBM cells to TMZ by suppressing HR- and MMEJ-mediated repair of TMZ-induced DSBs. Because no pharmacological inhibitors currently exist that can directly disrupt the interaction between RBBP4 and p300, we opted to inhibit RBBP4/p300-HAT activity using either a specific p300 inhibitor CCS1477 or the dual BRD4/p300 inhibitor NEO2734, both of which are either in preclinical or early phase clinical trials for various cancers.^28-32^ Our in vitro studies confirmed strong suppression of RAD51, C-MYC, and global H3K27 acetylation, along with enhanced TMZ sensitivity following treatment with CCS1477 or NEO2734, consistent with pharmacological blockade of RBBP4/p300-HAT activity.^17^ Intriguingly, GBM cells treated with TMZ in combination with CCS1477 or NEO2734 failed to efficiently repair TMZ-induced DSBs, as evidenced by elevated DNA damage response signaling and apoptosis. Collectively, these findings highlight the potential of targeting RBBP4/p300-HAT activity with either p300 or dual BRD4/p300 inhibitors as a novel therapeutic strategy in GBM.

We previously reported that a brain-penetrant CBP/p300 inhibitor, CPI-1612, potently reduced H3K27Ac and enhanced TMZ-induced DSBs in orthotopic GBM models.^17^ The efficacy studies, however, were terminated because of severe toxicity associated with this agent, either alone or in combination with TMZ. The CPI-1612 toxicity was unlikely related to the inhibitory effect on RBBP4/p300-HAT activity because, in this study, the inhibition of this complex by CCS1477 or NEO2734 significantly extended the survival of mice carrying the orthotopic GBM PDX tumors without causing any significant adverse effects on animal welfare. Instead, and intriguingly, the survival extension by either drug was comparable to the TMZ benefit, and the combination added a modest but significant survival benefit. This is the first demonstration of the potential of NEO2734 to penetrate through the BBB, but a previous report demonstrated that CCS1477 is a BBB-impermeable compound.^33^ Our LC-MS/MS analysis demonstrated significant concentrations of both CCS1477 and NEO2734 in non-tumor brain and enriched levels within intracranial tumor tissue, leading to suppressed H3K27Ac in vivo. These results confirmed that both agents could enter the brain at pharmacologically relevant levels. The current CCS1477 discrepancy with prior reports may be explained by differences in the dosing schedule, brain harvest timing, and the relatively permeable BBB associated with high-grade gliomas.^34^ Nonetheless, our LC-MS/MS findings require further validation by classical pharmacodynamics and pharmacokinetics studies.

In conclusion, we demonstrate for the first time the translational potential of inhibiting the RBBP4/p300 axis using CCS1477 or NEO2734, 2 clinically relevant and promising compounds. Both agents confer a survival benefit comparable to TMZ in a GBM43 orthotopic model, with a modest additional benefit observed upon combination treatment. However, further preclinical studies are required to validate these findings, including detailed pharmacokinetic and pharmacodynamic analyses to optimize the brain tumor distribution and exposure kinetics of CCS1477 and NEO2734 and to enhance their therapeutic synergy with TMZ in vivo.

## Supplementary Material

vdag141_Supplementary_Data

## Data Availability

All data generated for this study can be found within the manuscript or supplementary materials. Information related to the PDX lines can be found on the Mayo Clinic Brain Tumor Patient derived Xenograft National Resource website (https://www.mayo.edu/research/labs/translational-neuro-oncology/mayo-clinic-brain-tumor-patient-derived-xenograft-national-resource/pdx-characteristics/pdx-phenotype).
