# Supplementary material for: Pharmacologic inhibition of RBBP4/p300-mediated homologous recombination activity enhances glioblastoma sensitivity to temozolomide
Source: Neurooncol Adv. 2026 May 29;8(1):vdag141. doi: 10.1093/noajnl/vdag141 (PMC13264390; doi:10.1093/noajnl/vdag141)
Supplement: vdag141_Supplementary_Data [file vdag141_supplementary_data.pdf]

Supplementary Materials and Methods

Antibodies

For western blotting, the primary antibodies included RBBP4 Mouse mAb (Proteintech Cat# 66060-1-1g), p300 (E8S2V) Rabbit mAb (Cell Signaling Cat# 57625S), Rad51 (D4B10) Rabbit mAb (Cell Signaling Cat# 8875S), c-Myc (D84C12) Rabbit mAb (Cell Signaling Cat# 5605S), Acetyl-Histone H3 (Lys27) Rabbit Ab (Cell Signaling Cat# 4353S), Histone H3 (D1H2) XP® Rabbit mAb (Cell Signaling Cat# 4499S), Cleaved PARP (Asp214) (D64E10) XP® Rabbit mAb (Cell signaling Cat. # 5625S), anti-vinculin (E1E9V) XP(R) rabbit mAb (Cell Signaling cat# 13901S), beta-actin rabbit Ab (Cell Signaling Cat# 4967S), and secondary antibody was anti-Rabbit IgG, HRP-linked antibody (Cell signaling Cat. # 7074S) and anti-mouse IgG, HRP-linked antibody (Cell signaling Cat. # 7076S).

For γ-H2AX DNA damage foci, the primary antibody used was phospho-histone H2AX (S139) (20E3) rabbit mAb (Cell Signaling Cat# 9718S), and the secondary antibody was Alexa Fluor^{TM} 594 goat anti-rabbit IgG (H+L) (Invitrogen Cat# A11012).

DNA repair Activity Reporter Assay

Briefly, FM-HCR reporter plasmids were introduced into cells by lipid mediated transfection. After 24 hours incubation, cells were released by trypsinization, recovered by centrifugation, and resuspended in complete media for flow cytometric analysis. Fluorescent protein expression from double strand break repair reporter plasmids was normalized to fluorescent protein expression from a co-transfected plasmid that controlled for transfection efficiency. This normalized fluorescent protein expression was normalized to expression of a damage-free control that was similarly controlled for transfection efficiency. The detailed strategy for gating,

compensation, and calculating reporter expression for FM-HCR have been reported in a methodological manuscript [1].

Chromatin Immunoprecipitation (ChIP)

Tissue chromatin immunoprecipitation (ChIP) was performed using the Magna-ChIP™ G Tissue kit, Cat. # 17-20000 (Millipore, Billerica, MA). The GBM tissues were processed according to the protocol supplied by the vendor (Millipore). The antibodies used were anti-acetyl-lysine 27 histone H3 (H3K27ac rabbit monoclonal antibody, Diagenode Inc. Cat# C15210016) and Rb mAb to RbAp48 [EPR3411] (Abcam Cat# ab79416). The distal promoter region critical for RAD51 translation was targeted by a quantitative PCR using human-specific primer sequences: 5′-TCTTCTCGAGCTTCCTCAGC-3’ (forward), 5′-AGCGCTCTTGTGGTTTGTTT-3′ (reverse), and the ChIP enrichment was evaluated relative to the input chromatin. ChIP-seq data shown was from an independent study that we previously reported [2]. The access number to access this data is included in the main manuscript.

Liquid Chromatography-Mass Spectrometry

General: Unless otherwise specified, all reagents were obtained from commercial sources, were of the highest purity available and used without further purification. For all LC-MS/MS experiments LC-MS grade reagents and solvents were used. NEO-2734 (99.92% purity, catalog number S9648) and CCS-1477 (99.62%, catalog number S9667) were obtained from Selleck Chemicals.

Extraction from plasma samples: Frozen plasma samples were thawed on ice and mixed with a vortex mixer for 30 seconds prior to removal of a 50 µL of plasma to a sterile 1.5 mL centrifuge tube. To this tube, 200 µL of ice cold 50:50 (v:v) methanol:acetonitrile was added followed by

mixing for 30 seconds with a vortex mixer. This sample was centrifuged at 21,000 rcf at 4°C for 15 minutes. An aliquot of the supernatant was removed for analysis by LC-MS/MS.

Extraction from tissue samples: In a 2 mL cryovial, 500 µL of ice cold 50:50 (v:v) methanol:acetonitrile was added to a pre-weighed tissue sample and mixed with a vortex mixer for 30 seconds. The sample was then homogenized with a handheld homogenizer (Fisherbrand 150) equipped with a plastic disposable generator probe for three cycles, first for 10 seconds at the lowest setting (5,000 rpm), followed by two additional 15 second cycles at 15,000 rpm. The tube was capped and frozen by placing in a -80°C freezer for 1 hour. The frozen sample was then thawed on ice for 30 minutes, mixed with a vortex mixer for 30 seconds and frozen a second time. The frozen sample was thawed on ice for 30 minutes, mixed with a vortex mixer for 30 seconds and then centrifuged at 21,000 rcf at 4°C for 15 minutes. The supernatant was removed to a fresh sterile 1.5 mL microcentrifuge tube and again centrifuged at 21,000 rcf at 4°C for 15 minutes. An aliquot of the supernatant was removed for analysis by LC-MS/MS.

LC-MS/MS analysis: The liquid chromatography-mass spectrometry analysis was performed using a 6460C triple quad mass spectrometer (Agilent) with an inline 1290 LC system (Agilent) running a 2.1x100 mm C-18 column (Zorbax Eclipse Plus C18, 3.5 micron, Agilent). Chromatographic separation was achieved with an isocratic elution in 50% solvent A (10% acetonitrile in 10 mM ammonium acetate) and 50% solvent B (90% acetonitrile in 10 mM ammonium acetate) to 100% solvent B for 4 minutes followed by a linear gradient to 100% solvent B for 4 minutes followed by an isocratic elution in 100% gradient B for 4 minutes, at a flow rate of 0.25 mL/min. Using parent ion masses of 535.6 (M+H) and 436.5 (M+H) for CCS-1477 and NEO-2734, respectively, multiple reaction monitoring in positive ion mode with nitrogen as a nebulizing gas identified fragment ions at 81.1 (fragmenter voltage 135 V, collision energy 30 eV) and 350.9 (fragmenter voltage 135 V, collision energy 35 eV), respectively, with the largest signals. Further instrument optimization to maximize signal yielded the following experimental condition for mass spectrometer data collection: ion source temperature of 320°C,

ion source gas flow of 10 L/min, sheath gas flow of 11 L/min, nebulizer pressure of 25 psi, nozzle voltage of 500 V and capillary current of 3000 V. Standards were generated and analyzed for each drug using 6 concentrations spanning 32 nM through 100 µM. Peak areas in the chromatograms of samples and standards, run on the same day, were integrated (counts per second) using the Agilent QQQ Quantitative Analysis software (v 10.1) and a standard curve was generated using the standards data. Drug concentrations (plasma) or amounts (in moles/mg for tissues) were calculated from the respective standard curve.

H3K27 Acetylation Status by Immunofluorescence

To complement LC-MS/MS data, the *in vivo* effects of CCS1477 and NEO2734 on acetylation of lysine 27 of histone H3 (H3K27Ac) were assessed using the brain or flank tumors harvested two hours after the last dose from mice that were treated either with placebo, CCS1477, or NEO2734. Brains and flank tissues were fixed in 4% paraformaldehyde and embedded into optimal cutting temperature compound (OCT) and stored at -80°C. Frozen sections (5µm) were used for immunofluorescence staining following a previous published protocol [3]. The primary antibody used was anti H3K27Ac Rabbit Ab (Cell Signaling Cat# 4353S) and secondary antibody was Alexa Fluor™ 594 goat anti-rabbit IgG (H+L) (Invitrogen Cat# A11012). The slides were mounted in DAPI-containing antifade mounting solution (ThermoFisher) and immunofluorescence was visualized using a LSM900 confocal microscope (Zeiss). The fluorescence intensity was evaluated using ImageJ.

References

1. Fu D, Calvo JA, Samson LD. Balancing repair and tolerance of DNA damage caused by alkylating agents. *Nat Rev Cancer.* 2012; 12(2):104-120.

2. Kitange GJ, Mladek AC, Schroeder MA, et al. Retinoblastoma Binding Protein 4 Modulates Temozolomide Sensitivity in Glioblastoma by Regulating DNA Repair Proteins. *Cell Rep.* 2016; 14(11):2587-2598.

3. Mladek AC, Yan H, Tian S, et al. RBBP4-p300 axis modulates expression of genes essential for cell survival and is a potential target for therapy in glioblastoma. *Neuro Oncol.* 2022; 24(8):1261-1272.

Supplementary Figure 1

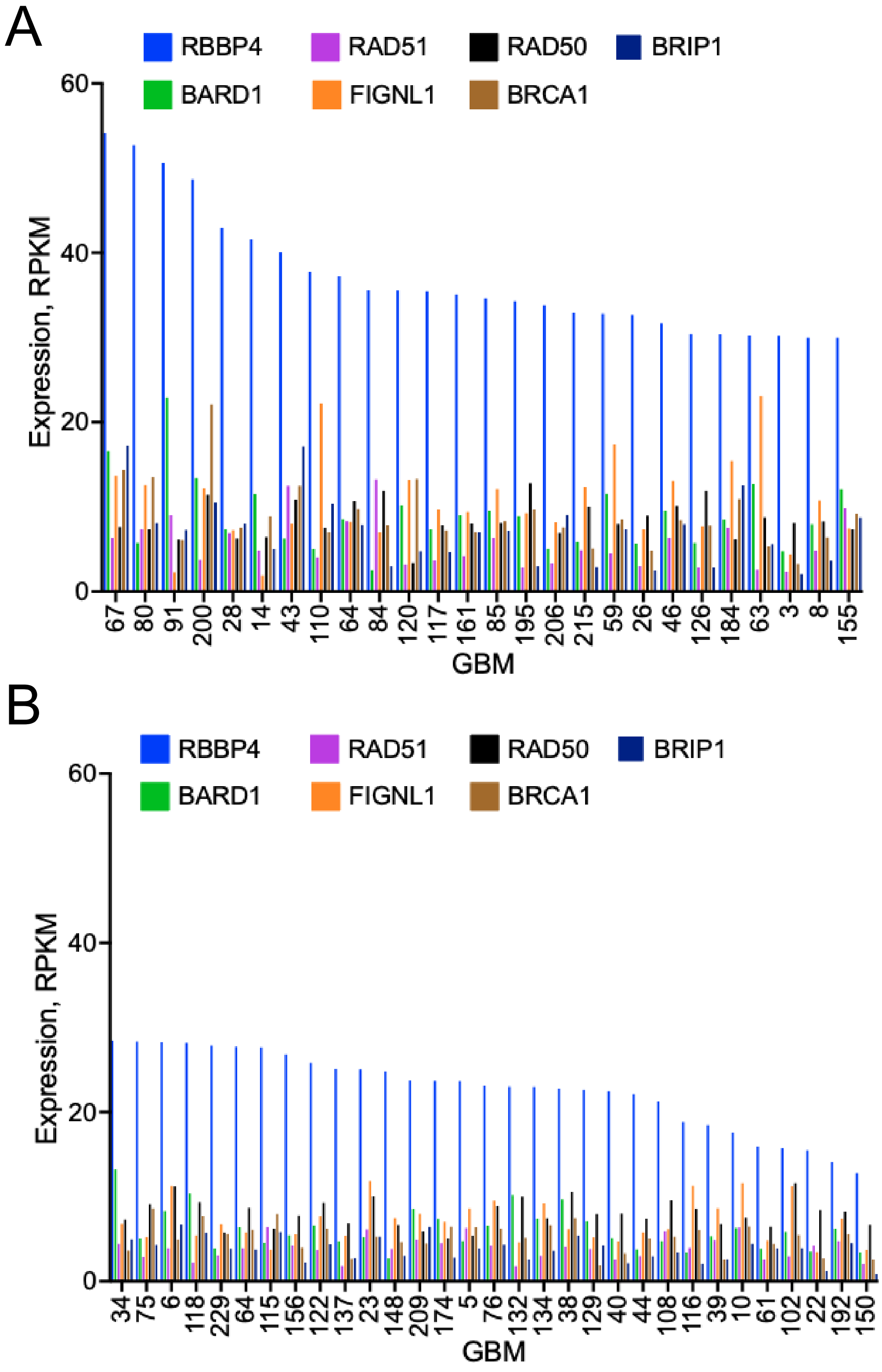

Supplementary Figure 1: Expression of RBBP4 and six HR repair gene transcripts across 58 GBM PDX models. RNA-seq data for the Mayo Clinic PDXs were obtained from the publicly available cBioPortal for Cancer Genomics (www.cbioportal.org). The PDXs were stratified into "RBBP4-high" and "RBBP4-low" groups using the mean expression across all 58 models as the cut-off. **(A)** Expression of the six HR genes and RBBP4 transcripts within RBBP4-high PDXs. **(B)** Expression of the six HR genes and RBBP4 within RBBP4-low PDXs. Overall, RBBP4-high PDXs displayed significantly higher transcript levels of the six HR genes compared to RBBP4-low PDXs.

Supplementary Figure 2

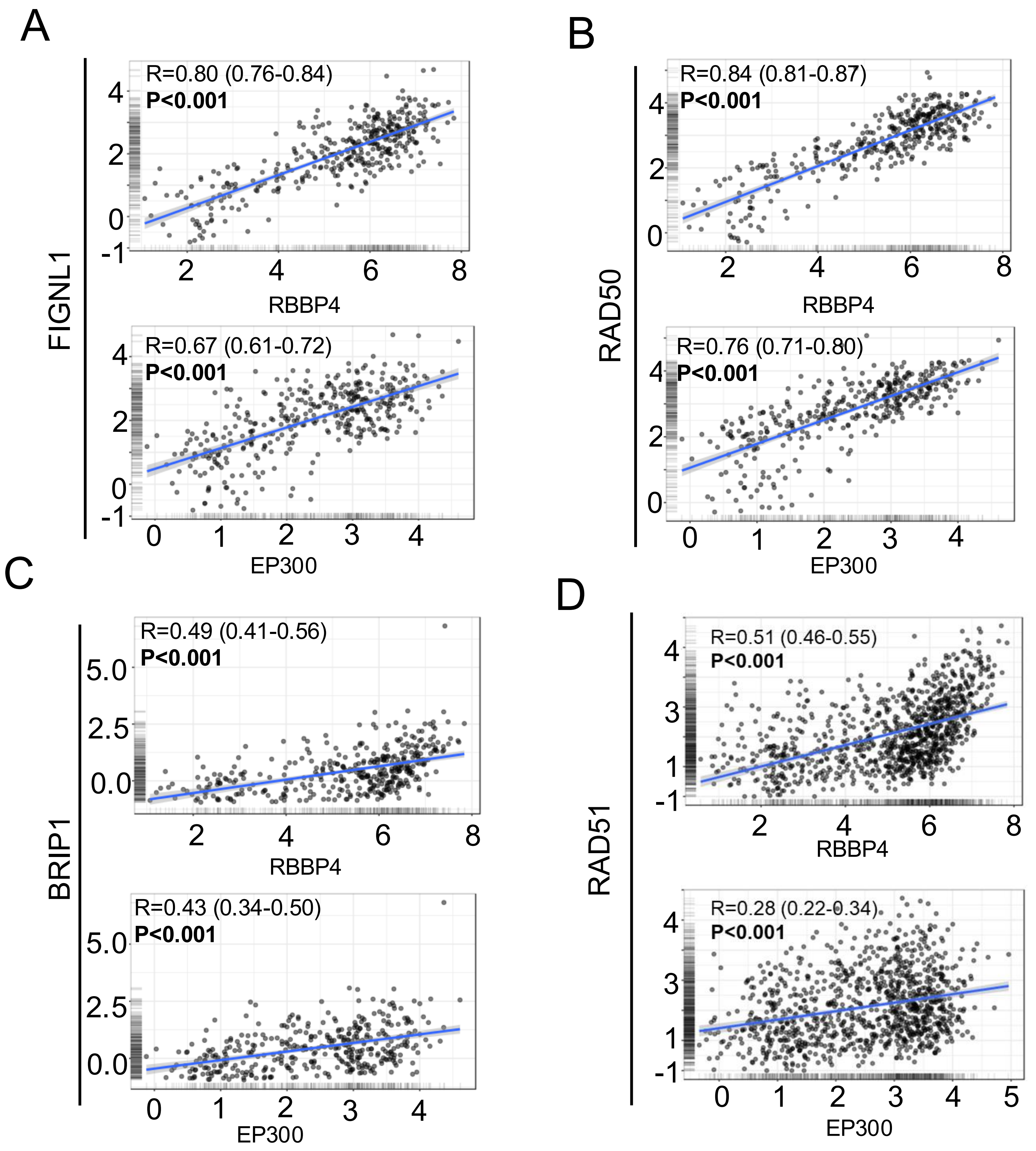

Supplementary Figure 2: Expression and correlation of RBBP4 and p300 with HR repair genes in GBM PDX models. Scatter plots showing the correlation between RBBP4 and p300 with HR repair gene pairs A) FIGNL1 B) RAD50 C) BRIP1 and D) RAD51. Pearson correlation coefficients (R) and p-values are displayed in each panel.

Supplementary Figure 3

A

CGGA Database

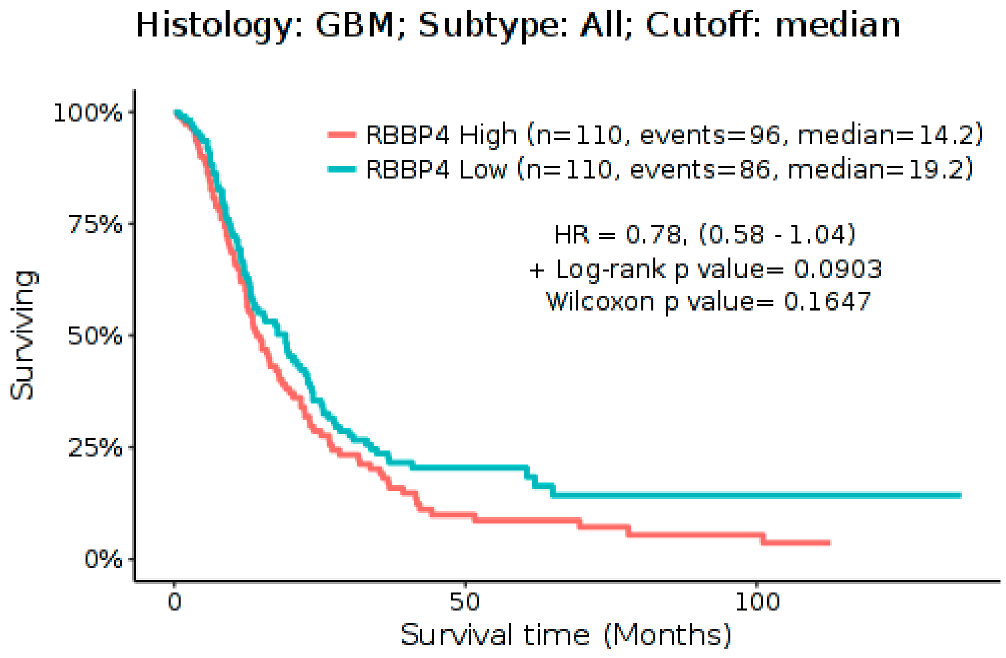

B

Rembrandt Database

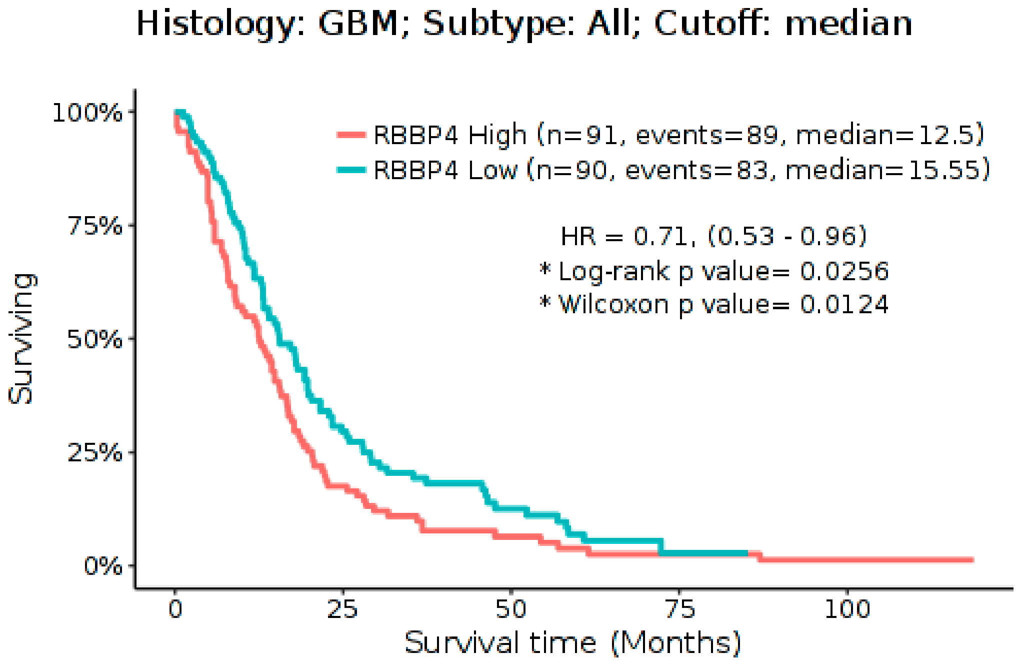

Supplementary Figure 3: RBBP4 expression correlates with GBM patient outcomes. The relationship between RBBP4 mRNA expression and the survival of GBM patients was analyzed using the **(A)** Chinese Glioma Genome Atlas (CGGA) and **(B)** Rembrandt databases. Overall, high RBBP4 expression is associated with poorer outcomes, with statistical significance observed in the Rembrandt patient cohort.

Supplementary Figure 4

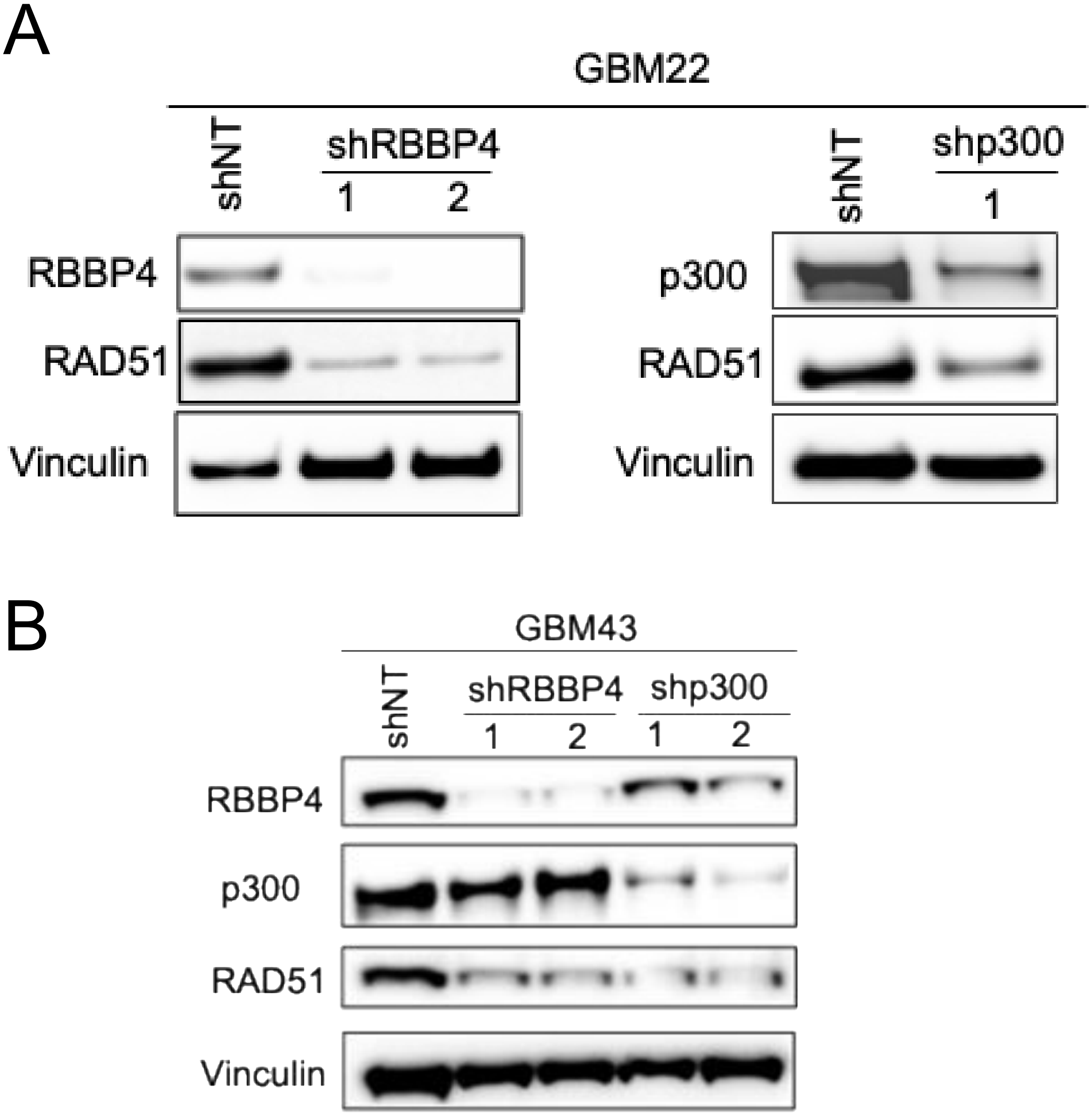

Supplementary Figure 4: Effects of RBBP4 or p300 shRNA silencing on RAD51 expression. GBM22 **(A)** and GBM43 **(B)** cells were transduced with lentiviral shRNA constructs targeting RBBP4 or p300. Following antibiotic selection, protein levels were evaluated to confirm knockdown efficiency and the subsequent effect on RAD51 expression. Vinculin was used as a loading control.

Supplementary Figure 5

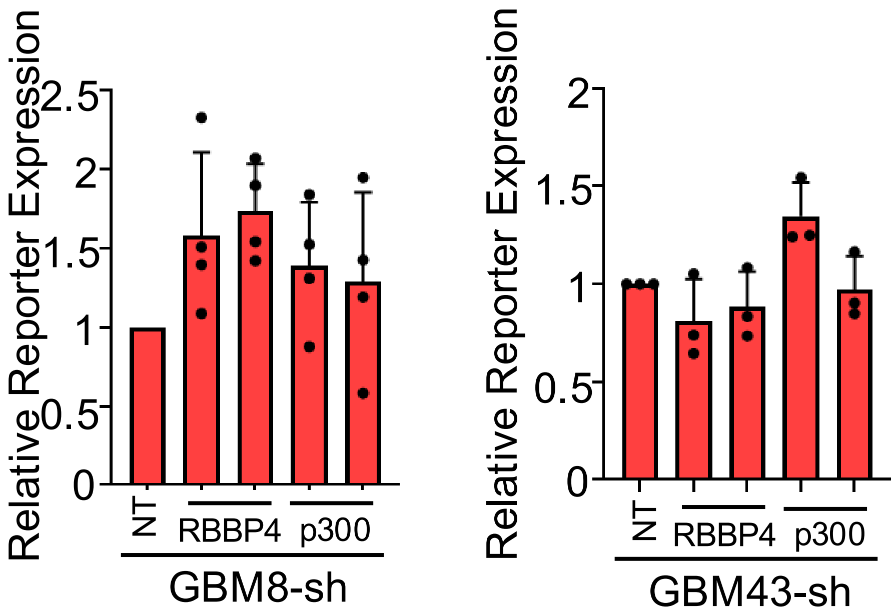

Supplementary Figure 5: Impact of silencing RBBP4 and p300 on NHEJ repair pathway. DNA damage repair reporter assays in GBM8 (right) and GBM43 (left) cells showing unchanged in classic NHEJ activity upon knocking down of either RBBP4 or p300. Data represents the mean ± SEM of three independent experiments.

Supplementary Figure 6

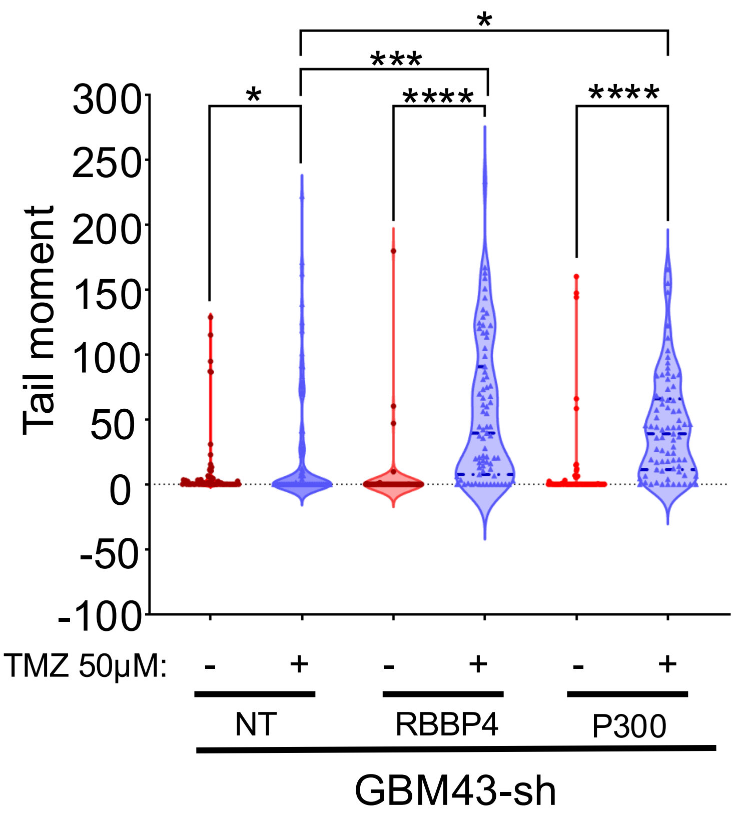

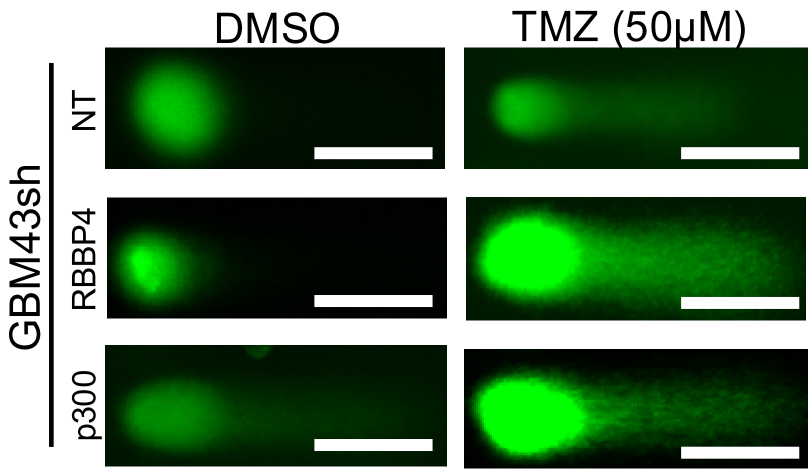

Supplementary Figure 6: Effect of RBBP4 and p300 silencing on the accumulation of TMZ-induced DSBs. Violin plots (upper panel) indicating increased DNA damage after treatment of GBM43-shNT, -shRBBP4 and -shp300 with either DMSO or 50 µM of TMZ. Representative comet assay images showing increased DNA fragmentation in shRBBP4 and shp300 GBM43 cells treated with TMZ (lower panel). Scale bar = 10 µm. Statistical significance is indicated as *p < 0.05, ***p < 0.01, ****p < 0.0001.

Supplementary Figure 7

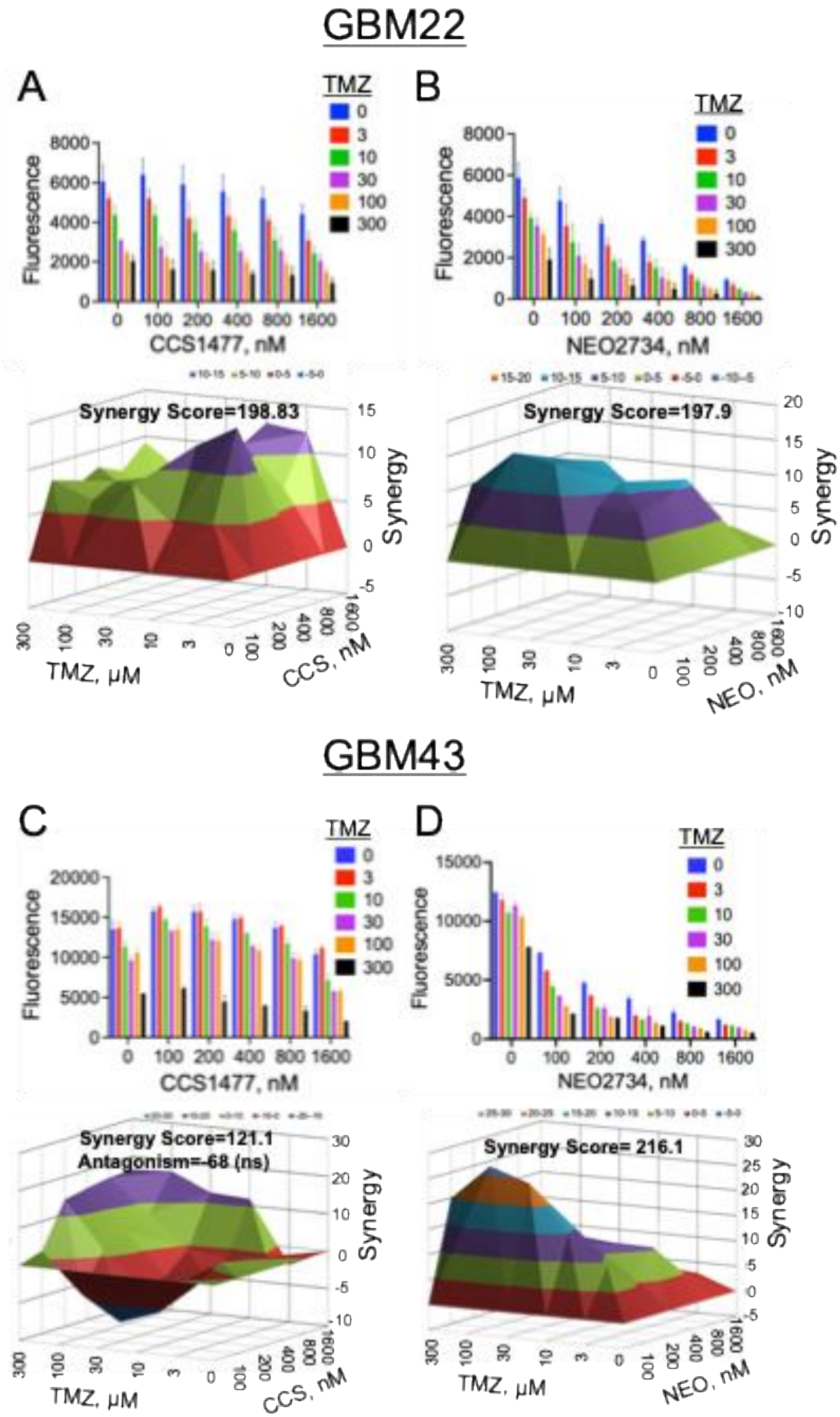

Supplementary Figure 7: Synergistic TMZ sensitization in GBM cells by CCS1477 and NEO2734. PDX GBM22 (A-B) and GBM43 (C-D) cells were treated with TMZ, with and without CCS1477 or NEO2734, and incubated under standard culture conditions for 7 days. Drug toxicity was evaluated using the CyQuant proliferation assay. Drug synergy was evaluated using MacSynergy II software; a synergy score of 100 or above was considered significant. Bar graphs represent the mean ± SEM of three independent experiments, each conducted in triplicate.

Supplementary Figure 8

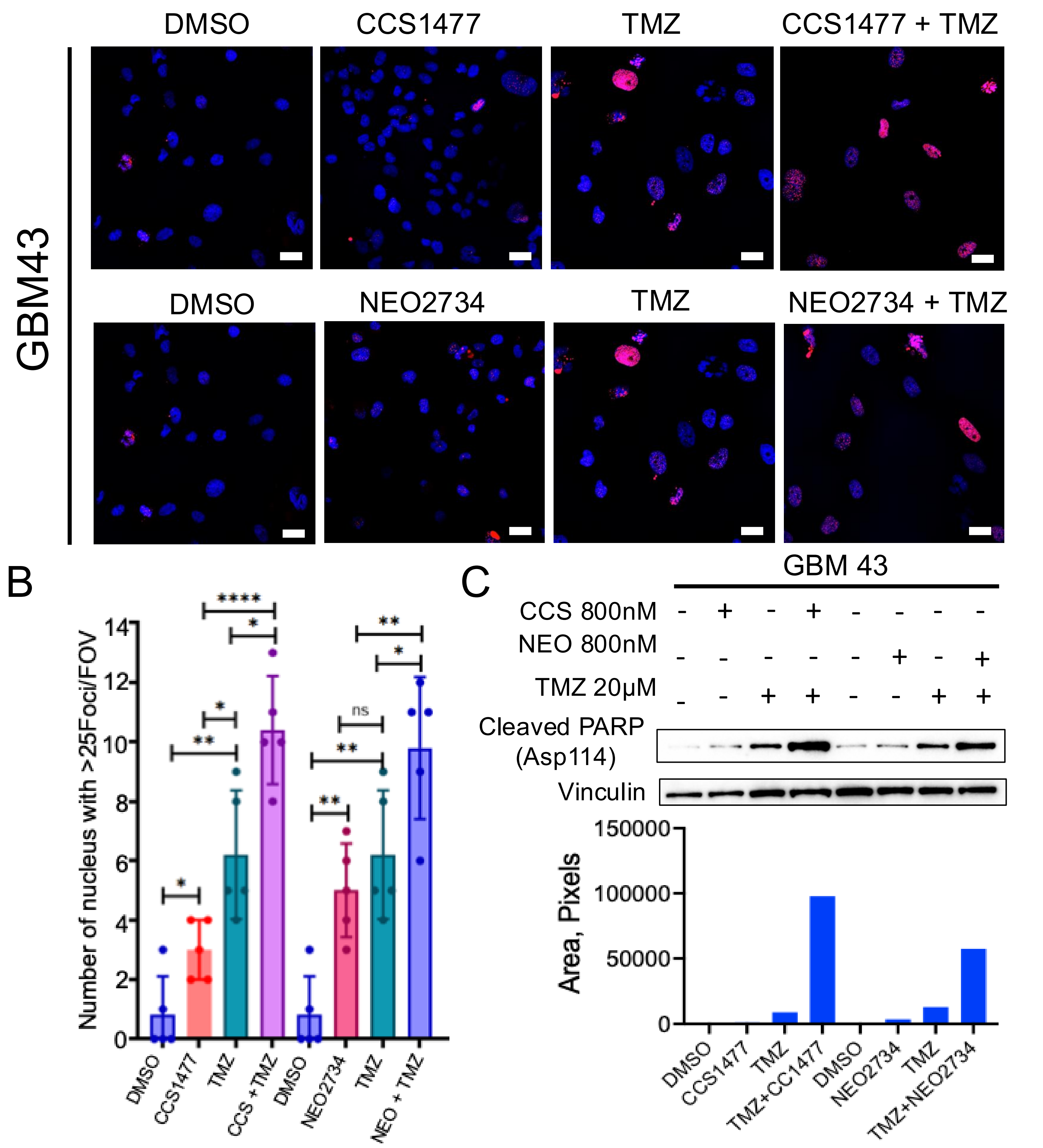

Supplementary Figure 8: Pharmacological inhibition of p300 kills GBM cells by apoptosis. (A) Evaluation of γH2AX foci in GBM43 cells treated with CCS1477, NEO2734, TMZ, and CCS1477+ TMZ or NEO2734 + TMZ. γ-H2A foci were detected using immunofluorescence staining performed 72 hours later (left panel). (B) The number of nuclei with ≥ 25 γ-H2AX foci was counted and graphed (right panel). (C) Upper panel is a representative of three independent Western blots showing an increase in cleaved PARP (Asp114) levels in GBM43 upon 72hrs treatment with the indicated concentrations of CCS1477, NEO2734, TMZ, and CCS1477+TMZ or NEO2734 + TMZ, and lower panel is the band quantification (B) Magnification bar = 20μM. Statistical significance is indicated as *p < 0.05, ** p-value <0.01, ***p-value <0.001.

Supplementary Figure 9

A

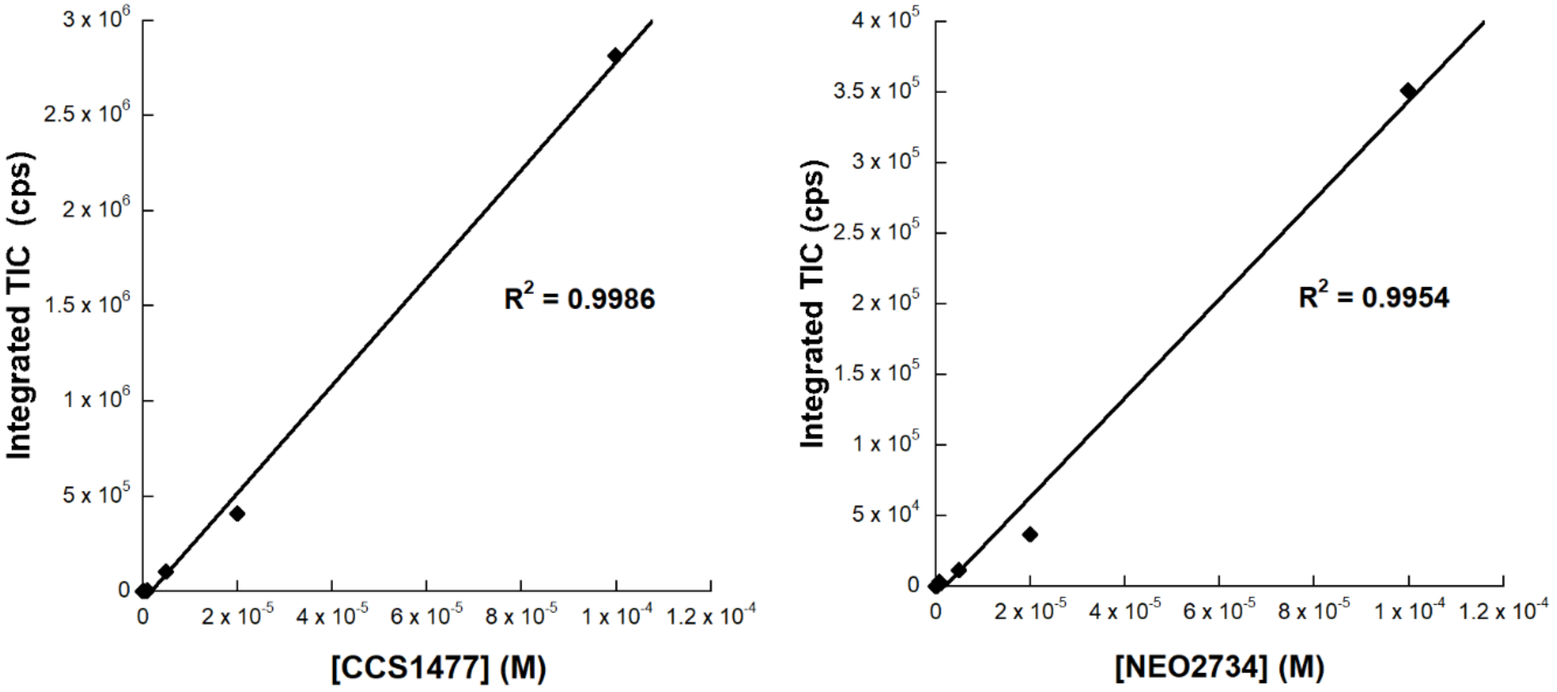

B

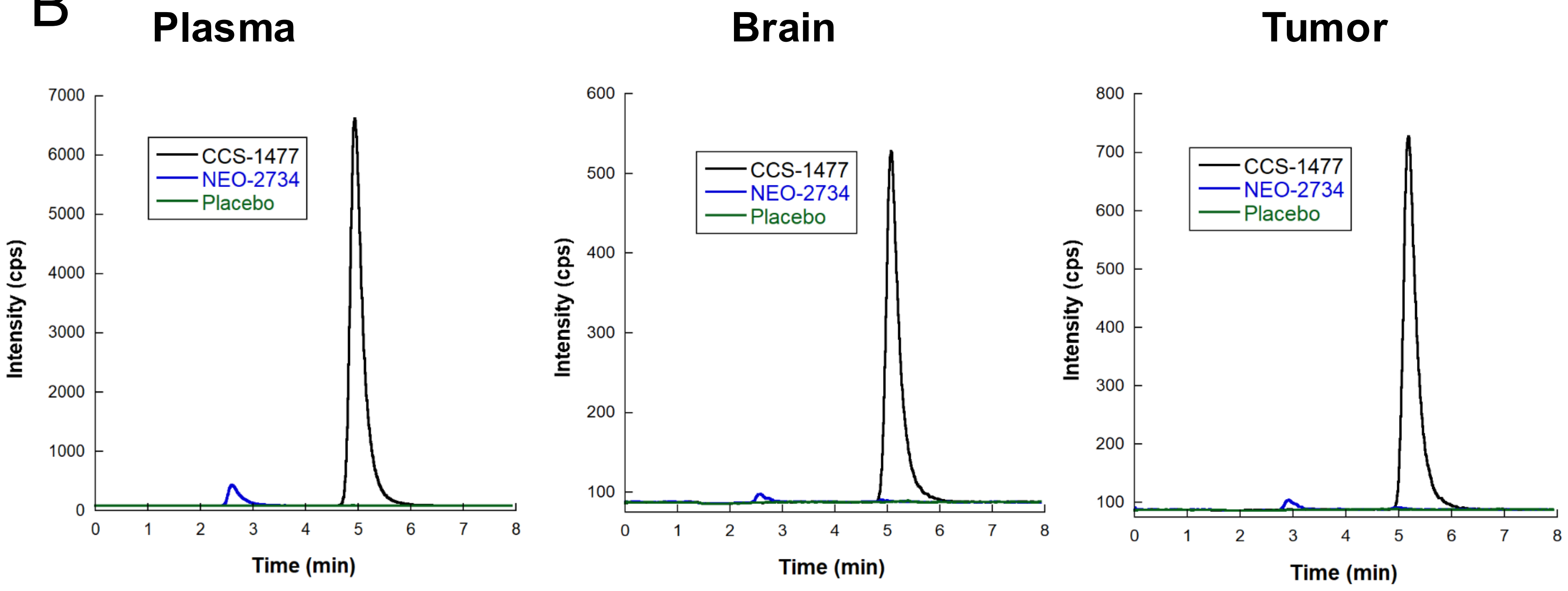

Supplementary Figure 9: LC-MS/MS showing distribution of CCS1477 and NEO2734 in plasma, brain, and IC tumors. (A) Standard curves generated for LC-MS/MS analysis of CCS1477 (left) and NEO2734 (right). Six concentrations ranging from 32nM to 100μM were analyzed as described in the methods section and the integrated intensity values (given in counts per second) were plotted against the concentrations. The R^2 values for the linear fit are shown on the graph. (B) Representative LC-MS/MS total ion chromatograms for methanol-chloroform extracts of plasma and tissue samples. Shown are the results of plasma (left), brain (center), and tumor (right) samples, 4 hours after treatment, for CCS-1477 (black curve), NEO-2734 (blue curve) and placebo (green curve) treated animals. Both drugs were observable at concentrations well above baseline in all samples. Note that the intensity difference observed between the two drugs is a result of the difference in intensity for the ions counted during analysis and does not correlate to a concentration difference.

Supplementary Figure 10

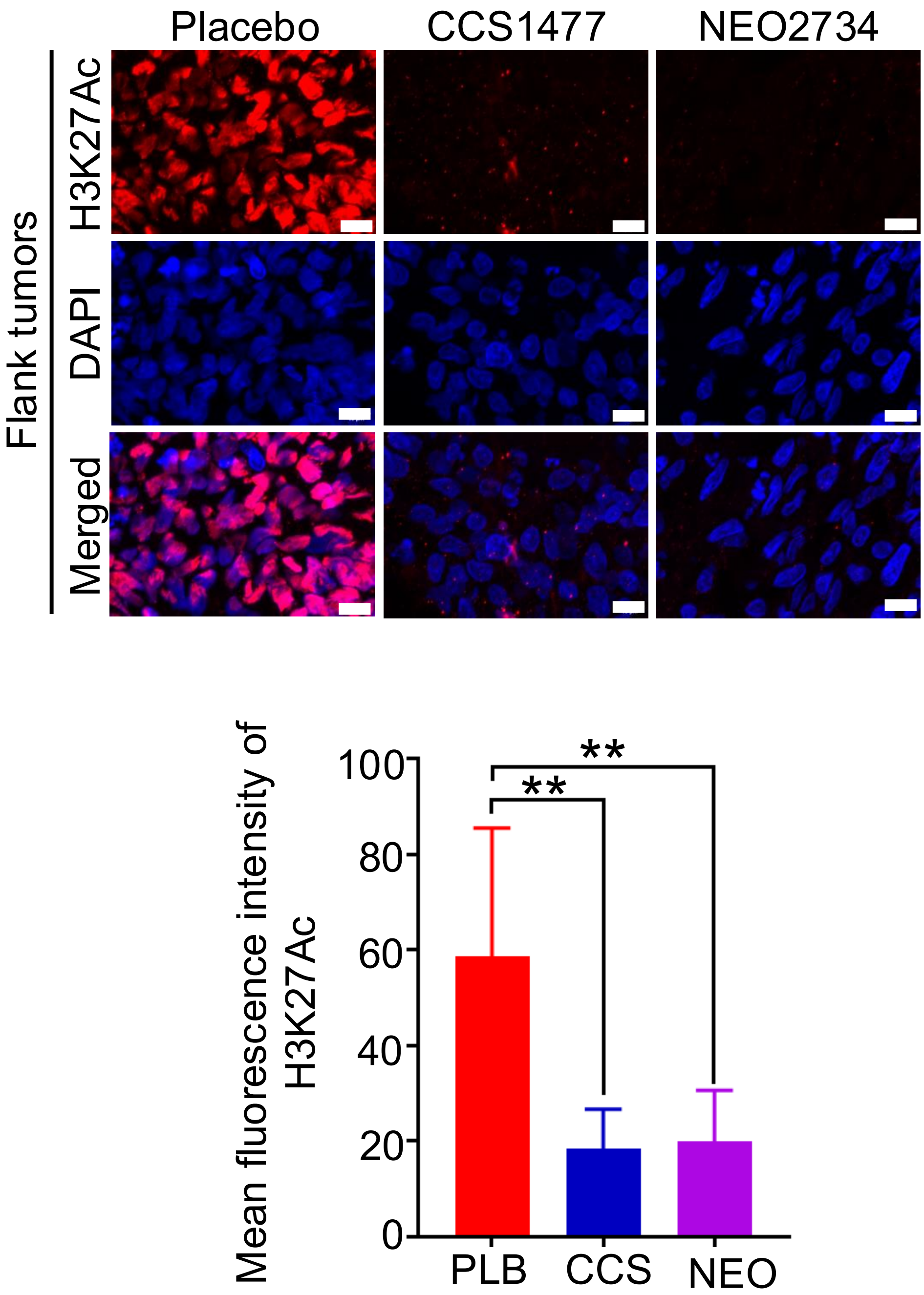

Supplementary Figure 10: Effect of p300 inhibition on in vivo in flank tumors. Representative images from PD studies showing H3K27ac in mice bearing GBM43 flank tumors after treatment with placebo, 20mg/kg CCS1477 or 10mg/kg NEO2734 once daily for 5 days with tumors harvested 2 hours after the last dose (scale bars = 20 μm). Bar graphs indicate the mean fluorescence intensity between the different treatment groups. Statistical significance is indicated as ** p-value <0.01

Supplementary Figure 11

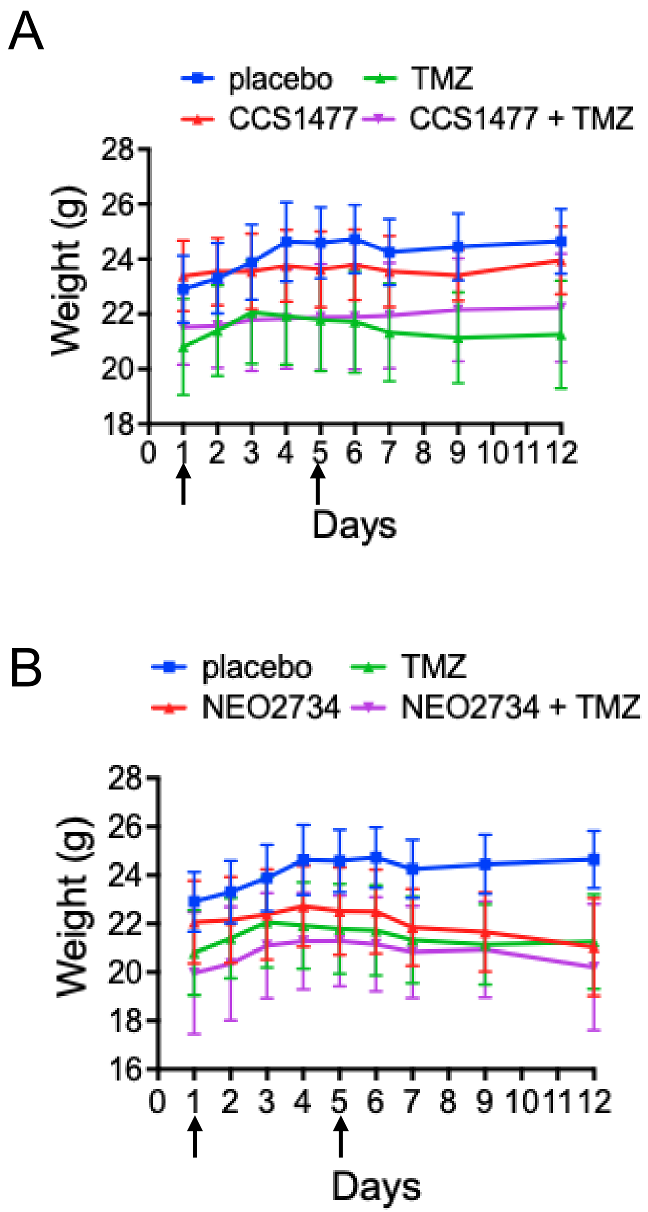

Supplementary Figure 11: Effect of CCS1477 and NEO2734 on animal body weights. Animals treated with TMZ, CCS1477, or NEO2734, either as monotherapies or in combination, were monitored via daily weight measurements. Plots were generated using GraphPad Prism software. (A) TMZ and/or CCS1477 and (B) TMZ and/or NEO2734. “0” denotes day 10 after intracranial injections; the TMZ and placebo arms are shared for both panels; Arrows= treatment starting and end points.
